# Neonatal intestinal mucus barrier changes in response to maturity, inflammation, and sodium decanoate supplementation

**DOI:** 10.1038/s41598-024-58356-5

**Published:** 2024-04-01

**Authors:** Janni Støvring Mortensen, Søren S.-R. Bohr, Lasse Skjoldborg Krog, Johan Peter Bøtker, Vaya Kapousidou, Lasse Saaby, Nikos S. Hatzakis, Hanne Mørck Nielsen, Duc Ninh Nguyen, Stine Rønholt

**Affiliations:** 1 Center for Biopharmaceuticals and Biobarriers in Drug Delivery (BioDelivery), Department of Pharmacy, Faculty of Health and Medical Sciences, University of Copenhagen, Universitetsparken 2, 2100 Copenhagen, Denmark; 2 Department of Chemistry and Nanoscience Center, Faculty of Science, University of Copenhagen, Universitetsparken 5, 2100 Copenhagen, Denmark; 3 Department of Pharmacy, Faculty of Health and Medical Sciences, University of Copenhagen, Universitetsparken 2, 2100 Copenhagen, Denmark; 4 Bioneer A/S, Kogle Allé 2, 2970 Hørsholm, Denmark; 5 NovoNordisk Center for Protein Research, Faculty of Health and Medical Sciences, University of Copenhagen, Blegdamsvej 3B, 2200 Copenhagen, Denmark; 6 Department of Veterinary and Animal Sciences, University of Copenhagen, Dyrlægevej 68, 1870 Frederiksberg C, Denmark

**Keywords:** Mucus diffusivity, Intestinal barrier, Neonatal, Sodium caprate supplement, Necrotizing enterocolitis, Perinatal asphyxia, Biological techniques, Biophysics, Biotechnology, Developmental biology, Gastroenterology, Medical research

## Abstract

The integrity of the intestinal mucus barrier is crucial for human health, as it serves as the body's first line of defense against pathogens. However, postnatal development of the mucus barrier and interactions between maturity and its ability to adapt to external challenges in neonatal infants remain unclear. In this study, we unveil a distinct developmental trajectory of the mucus barrier in preterm piglets, leading to enhanced mucus microstructure and reduced mucus diffusivity compared to term piglets. Notably, we found that necrotizing enterocolitis (NEC) is associated with increased mucus diffusivity of our large pathogen model compound, establishing a direct link between the NEC condition and the mucus barrier. Furthermore, we observed that addition of sodium decanoate had varying effects on mucus diffusivity depending on maturity and health state of the piglets. These findings demonstrate that regulatory mechanisms governing the neonatal mucosal barrier are highly complex and are influenced by age, maturity, and health conditions. Therefore, our results highlight the need for specific therapeutic strategies tailored to each neonatal period to ensure optimal gut health.

## Introduction

In the gastrointestinal tract, the mucus layer serves as innate protection for the intestinal epithelium, guarding it against invasion by both commensal and pathogenic bacteria present in the intestinal lumen. Therefore, the integrity of the intestinal mucus barrier is of paramount importance for maintaining the health of the local gut microbiota and, more importantly, for overall systemic health^1–3^. However, in preterm infants (born before 37 weeks of gestation), the intestinal mucus barrier is underdeveloped. These infants often exhibit low mucin production and insufficient mucus thickness, which hinders their ability to effectively trap bacteria and toxic macromolecules. Therefore, preterm infants are more susceptible to inflammation and infections^4,5^. This vulnerability contributes to a higher risk of infectious diseases, including necrotizing enterocolitis (NEC), a severe inflammatory condition associated with high mortality rates^6–8^. Furthermore, complications that occur before and during delivery, such as perinatal asphyxia (PA), can further impede normal postnatal organ development and increase the likelihood of developing NEC^9,10^. However, despite the challenges faced by both preterm and term infants, our understanding of how the intestinal epithelial barrier and the mucus barrier develop after birth and whether these mucosal barrier properties are influenced by preterm complications (e.g., PA and NEC) remains limited. In turn, this knowledge gap poses difficulties in establishing effective dietary and treatment recommendations. Therefore, gaining a deeper understanding of the developmental trajectories of these barriers will provide valuable information for exploring alternative preventive medicine strategies for this vulnerable patient group.

Animal models play a crucial role in providing a fundamental understanding of the biological and biophysical properties of the mucus layer, and preterm piglets are widely acknowledged as one of the most clinically relevant models for studying preterm infants, by having similar birth weight, physiology and metabolism to that of preterm infants. Furthermore, preterm piglets are the only preterm animal model that can mimic the NEC and infection sensitivity in preterm infants. The immaturity of the systemic immune system in newborn piglets is very similar to that in preterm infants with poor response to ex vivo challenge and impaired functions of phagocytes^11–13^. Additionally, the structural and functional similarities between intestinal mucus from pigs and human intestinal mucus further strengthen the relevance of using preterm piglets as a model^14^.

The intestinal mucus barrier undergoes constant shedding and clearance, and the continuous renewal of the mucus layer is vital to maintaining intestinal barrier homeostasis. Goblet cells are responsible for producing and secreting highly *O-*glycosylated glycoproteins known as mucins, which are the main structural components of mucus^1,3^. Previous studies have demonstrated that preterm piglets exhibit significant differences in goblet cell density compared to term piglets^15^. This suggests a compromised mucus barrier in preterm piglets, explaining the higher risk of NEC and intestinal inflammation in preterm animals. Therefore, enhancing the neonatal mucus barrier could potentially reduce the risk of NEC. In studies involving young mice and pigs, supplementing the diet with sodium decanoate (C10), a common medium-chain fatty acid found in milk and infant formula^16,17^, has been reported to improve the overall growth performance and barrier function of the gastrointestinal tract in the supplemented animals^18,19^. However, the exact mechanisms by which C10 enhances the barrier properties of the gastrointestinal tract remain unknown. A previous study has identified another medium-chain fatty acid that improves the barrier properties of intestinal mucus in pigs^20^, leading to speculation whether C10 might also enhance the neonatal mucus barrier, thus explaining the observed improvements in the gastrointestinal tract’s barrier function^18,19^.

The present study provides the first biophysical characterization of the small intestinal mucus barrier in preterm, near-term, and term piglets during the first weeks of life, both with and without NEC or PA. The mucus barrier in preterm piglets follows a distinct developmental trajectory, which was different from that of term piglets. Interestingly, at postnatal day 5, mucus from preterm piglets exhibited an enhanced network structure and lower diffusivity compared to mucus from near-term and term piglets. However, preterm piglets that developed NEC displayed significantly increased mucus diffusivity of the large pathogen model compound at postnatal day 5, indicating that initial differences in mucus barrier properties may play a role in governing NEC onset.

The effects of C10 were highly dependent on the health condition and maturity of the piglets. For near-term piglets with PA, mucus diffusivity decreased in the presence of C10, whereas the opposite occurred in mucus from healthy preterm piglets. Conversely, C10 had no significant effect on mucus from healthy near-term piglets and preterm piglets with NEC. The extensive dataset generated in this study links the structural complexity of the neonatal porcine intestinal mucus to its barrier properties, thus providing invaluable insights into the physiology of the neonatal mucus barrier. In turn, these findings may serve as a basis for the development of novel therapeutic strategies for managing postnatal complications in newborn infants.

## Results

### The small intestinal mucus barrier exhibits dramatic changes during early life

**Figure 1:**
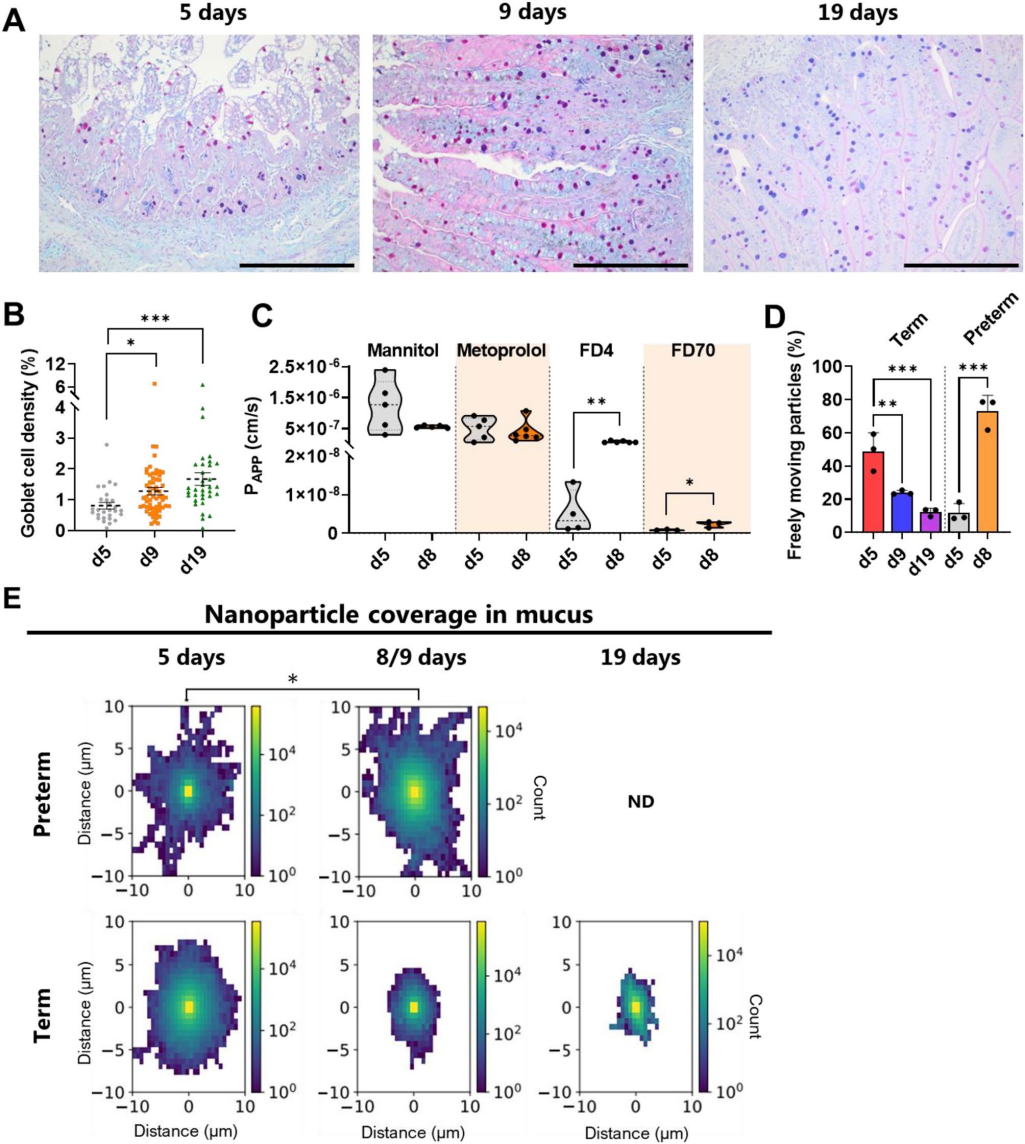
Effect of age on goblet cell density and nanoparticle movement in ex vivo mucus from healthy preterm and term piglets. (**A**) Representative micrographs of Alcian blue and periodic acid-Schiff (AB-PAS) stained distal small intestine (scale bar: 500 µm) and (**B**) counted goblet cell density at postnatal days 5, 9, and 19 in healthy preterm piglets (N = 36, 77 and 36, respectively). (**C**) Apparent permeability coefficients (P_App_ values) for ^14^C-mannitol, ^3^H-metoprolol, fluorescein-isothiocyanate dextran 4 kDa (FD4), and fluorescein-isothiocyanate dextran 59–77 kDa (FD70) permeation through ex vivo small intestinal mucosa from healthy preterm piglets at postnatal days 5 and 8 (N = 3–6, n = 1–2). (**D**) Fraction of 100–300 nm polystyrene nanoparticles moving freely in mucus (anomalous diffusion parameter, i.e., alpha value, α > 0.5). (**E**) 2D heatmap of average (98% quantile) nanoparticle coverage in mucus collected from healthy preterm or term piglets on postnatal days 5, 8/9, and 19, as obtained using single particle tracking (N = 3, n = 1–2). The black circles indicate data obtained from individual piglets. Data in the bar plots are presented as means with standard deviation. The variation in the number of piglets used for the mucosa permeability study was due to the study design and the exclusion of some data points that were below our lower limit of quantification. Statistically significant data are indicated as follows: *(*p* < 0.05), **(*p* < 0.01), ***(*p* < 0.001). The exact *p* values are listed in the Supplementary Information Table S4. The postnatal age of the piglets are given in days (e.g., d5 = 5 days old). *ND* not determined. All source data are provided as a Source Data file.

The mucus barrier plays a vital role in maintaining the homeostasis of the entire gastrointestinal tract, yet little is known regarding the properties and functions of the mucus barrier in preterm infants. To gain insights into the complexity of neonatal mucus development after birth, we initially characterized properties related to mucus production in preterm piglets at different postnatal ages. The density of mucin-containing goblet cells, assessed using alcian blue and periodic acid Schiff (AB-PAS) staining, serves as a surrogate marker for a healthy developing mucus layer^1,3,15^. Goblet cell density exhibited an increasing representation from 5 to 19 days after birth for preterm piglets (Fig. 1A, B). Furthermore, we observed that the post-translational modifications (PTMs) of synthesized mucins trended toward becoming more acidic, i.e., sulfur and carboxylic PTMs (stained blue), with increasing postnatal age (5 days vs. 19 days). However, neutral PTMs (stained magenta) appeared to be more predominant at 9 days (Fig. 1A)^21^. Changes in mucin quantity and PTM structure are known to impact the barrier properties of mucus^22–24^. To investigate whether the postnatal mucin changes (Fig. 1A, B) led to altered barrier properties of neonatal mucus, we introduced 100–300 nm nanoparticles (NPs) to the mucus. Using a single particle tracking assay, we directly observed the spatiotemporal localization of individual NPs, gaining insights into their movement within mucus^20,25^. In this study, NPs were employed as a model compound for large pathogens due to their comparable size to pathogenic microbes, such as viruses and bacteria^26^.

To understand the behavior of NPs in the mucus, we first calculated the diffusion coefficient from the trajectory of each NP (Supplementary Information Fig. S1 and Table S1). Afterward, we determined the average coverage of a considerable number of NPs (ranging from 4781 to 15,175) in the mucus and visualized the obtained data using coverage plots (Fig. 1E). Additionally, we calculated the anomalous diffusion parameter (alpha), which describes the confinement of NPs in the mucus (see Supplementary Information Fig. S1 for distributions).

The fraction of freely moving NPs (i.e., exhibiting movement similar to that in buffer^20^) decreased with increasing postnatal age for term piglets, while for preterm piglets, it increased sevenfold with increasing postnatal age (5 days vs. 8 days) (Fig. 1D). This was consistent with the increased NP coverage observed for 8-day-old preterm piglets (Fig. 1E), suggesting that at this postnatal age for preterm piglets, the mucus barrier might become more permeable to microbial penetration.

To assess whether the decreased mucus barrier properties in the 8-day preterm piglets also affected the barrier properties of the entire intestinal mucosa, we measured the permeation of model compounds through isolated mucosa as a function of time (see Supplementary Information Fig. S2 for flux curves). In this study, we used model compounds with varying physicochemical properties. Mannitol (182.2 Da, log *P* < – 2.5) and metoprolol (267.4 Da, log *P* > 1.9) served as small molecular hydrophilic and hydrophobic model compounds, respectively. Fluorescein-isothiocyanate dextran 4 kDa (FD4) and 59–77 kDa (FD70) were used as hydrophilic macromolecules, with sizes comparable to peptides (FD4)^27,28^ and small pathogens such as viruses (FD70)^26–29^. The apparent permeability coefficients (P_APP_), which describe the steady-state passive permeation of our model compounds, exhibited a direct correlation between the barrier properties of the mucus (Fig. 1D, E) and the mucosa's permeability to the macromolecules FD4 and FD70 (Fig. 1C). Taken together, these findings suggest that 8-day preterm piglets have a more permeable intestinal mucosa barrier than the 5 days preterm piglets.

Overall, we found that few days of difference in postnatal age between term and preterm piglets resulted in significant changes in the barrier properties of intestinal mucus. It should be noted that the observed trajectories may have been affected by differences in diets, as the 5 days preterm piglets were fed with another infant formula than the 8/9 and 19 days preterm piglets, and the term piglets were raised on the sow milk. Yet in clinical setting preterm and term infants would also receive different diets to cover their nutritional needs.

### Maturity at birth and rearing conditions significantly affect the properties of intestinal mucus

**Figure 2:**
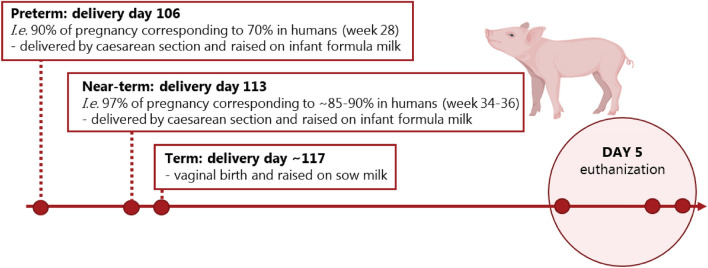
Schematic overview of birth maturity and rearing of 5-day old healthy piglets. Created with Biorender.com.

The impact of increasing postnatal age on mucus barrier properties differed between term and preterm piglets, suggesting that the birth maturity of the piglets is a contributing factor that influences the mucus barrier's behavior. To explore this further, additional analyses were conducted to examine the effect of piglet birth maturity on mucus properties. In contrast to the term piglets, all of which were delivered vaginally and raised by the sow, the preterm piglets were delivered via cesarean section and thereafter nourished with infant formula milk. Mode of delivery and rearing conditions are factors known to have significant effects on intestinal development^10,11,30^, which may account for the observed differences in Fig. 1D, E. To address these distinctions, we included a near-term group in our study. These near-term piglets were similar to the term piglets in terms of birth maturity but were delivered and reared under conditions similar to those of the preterm piglets, though the specific infant formula that was used varied between the preterm and near-term piglets to meet their nutritional needs (Fig. 2).

Interestingly, both the coverage and diffusion coefficient of the NPs in mucus were 3.5-fold higher for the near-term piglets compared to preterm piglets (Table 1). Additionally, in mucus from near-term piglets, half of the tracked NPs exhibited free movement, whereas the NPs in mucus from preterm piglets were significantly more confined, with only 12% of the tracked NPs moving freely (Table 1). Consequently, a mere 7-day difference in the birth maturity of the piglets had a substantial impact on the NPs' movement in mucus. Moreover, we found that the diffusion coefficients of the NPs in mucus were 2.5-fold higher (*p* = 0.003) in the near-term piglets compared to term piglets. Despite the faster moving NPs in mucus from near-term piglets than term piglets, the fractions of freely moving, confined and immobile NPs were found similar for the near-term and term piglets. This suggests that the NPs in mucus were equally affected by the porous network of mucus for both near-term and term piglets, but that for mucus from near-term piglets, the NPs moved faster within the pores. This observation suggests that the differences in birth delivery and rearing conditions, such as diet and living conditions, indeed lead to alterations in the intestinal mucus barrier.

**Table 1:** Schematic overview of how birth maturity and the rearing conditions of 5-day old healthy piglets influenced nanoparticle movement.

	Term	Near-term	Preterm
Nanoparticle coverage (µm^2^)	2.4 ± 0.5	3.5 ± 0.9	1.2 ± 0.7 (*p* = 0.0165)
Diffusion coefficients (× 10^–2^ µm^2^ s^−1^)	5.7 ± 1.2 (p = 0.0029)	13.3 ± 2.3	3.5 ± 1.0 (p = 0.0007)
Fraction of freely moving nanoparticles (α > 0.5) (%)	48.8 ± 11.3	51.0 ± 11.8	11.9 ± 5.5 (*p* = 0.0355)
Fraction of confined nanoparticles (0.001 < α < 0.5) (%)	49.6 ± 10.8	46.5 ± 10.7	83.1 ± 5.2 (*p* = 0.0071)
Fraction of immobile nanoparticles (α < 0.001) (%)	1.6 ± 0.8	2.5 ± 0.1	4.8 ± 0.5 (*p* = 0.0071)

Nanoparticle coverage, diffusion coefficients, and fraction of freely moving, confined, and immobile 100–300 nm polystyrene nanoparticles obtained using single particle tracking in mucus from term, near-term, or preterm born piglets postnatal day 5 (N = 3; n = 2). All data are presented as means with standard deviation. The data were statistically different from the near-term group, as indicated by the *p* values. All source data are provided as a Source Data file.

**Figure 3:**
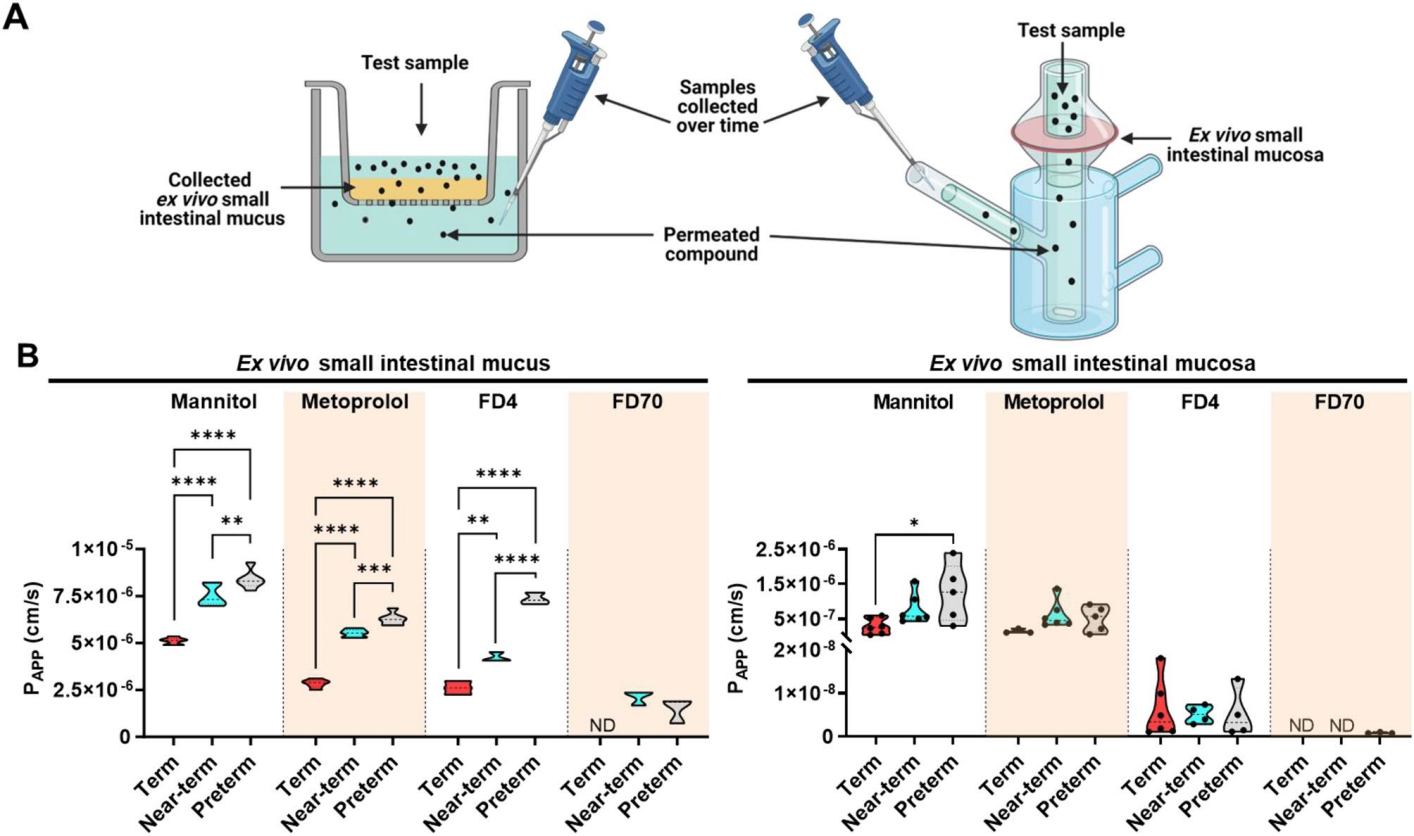
Apparent permeability coefficients (P_APP_) of model compounds through either isolated ex vivo small intestinal mucus or entire mucosa collected from 5-day old healthy piglets with different birth maturities. (**A**) Illustration of the filter insert method and the Franz diffusion cells, which were used for the mucus and mucosa permeability studies, respectively. Created with Biorender.com. (**B**) Obtained P_App_ values of ^14^C-mannitol, ^3^H-metoprolol, fluorescein-isothiocyanate dextran 4 kDa (FD4), and fluorescein-isothiocyanate dextran 59–77 kDa (FD70) through ex vivo small intestinal mucus (N = 1, n = 3–6) or the entire mucosa (N = 3–6, n = 1–2). Due to low sample volumes for the mucus study, mucus from a minimum of three piglets from each group was pooled and mixed, after which technical replicates were analyzed. The black circles indicate the data obtained from individual piglets. The variation in the number of piglets used for the mucosa permeability study was due to the study design and the exclusion of some data points that were below our lower limit of quantification. ND = not detected. For (**B**), one outlier was identified for FD4 through the mucosa of near-term piglets and was therefore not included in the presented data. Statistically significant data are indicated as follows: *(*p* < 0.05), **(*p* < 0.01), ***(*p* < 0.001), or ****(*p* < 0.0001). The exact *p* values are listed in Supplementary Information Table S4. Source data are provided as a Source Data file.

Next, we investigated whether the birth maturity of healthy piglets would also affect bulk mucus permeability and mucosa permeability (Fig. 3A). As illustrated in Fig. 3B, the calculated P_APP_ values were obtained from the permeability studies conducted on mucus and mucosa (flux curves are provided in Supplementary Information Fig. S3).

Permeation through mucus often decrease with increasing molecular weight and hydrophobicity of the model compounds (Fig. 3B). However, birth maturity significantly influenced the P_APP_ values for the individual model compounds. Specifically, the permeation of mannitol, metoprolol, and FD4 decreased with increasing birth maturity (i.e., term < near-term < preterm). Similarly, permeation through the mucosa was also influenced by the hydrophobicity and molecular weight of the permeating compound. The decreases in P_APP_ values for metoprolol, FD4, and FD70 compared to mannitol were two–sixfold, 129–576-fold, and 1502-fold, respectively (Supplementary Information Table S2). Although no statistically significant differences related to birth maturity were observed for mucosa permeability, term piglets tended to have lower mucosa permeability compared to near-term and preterm piglets, which was consistent with the observations regarding mucus permeability (Fig. 3B).

**Figure 4:**
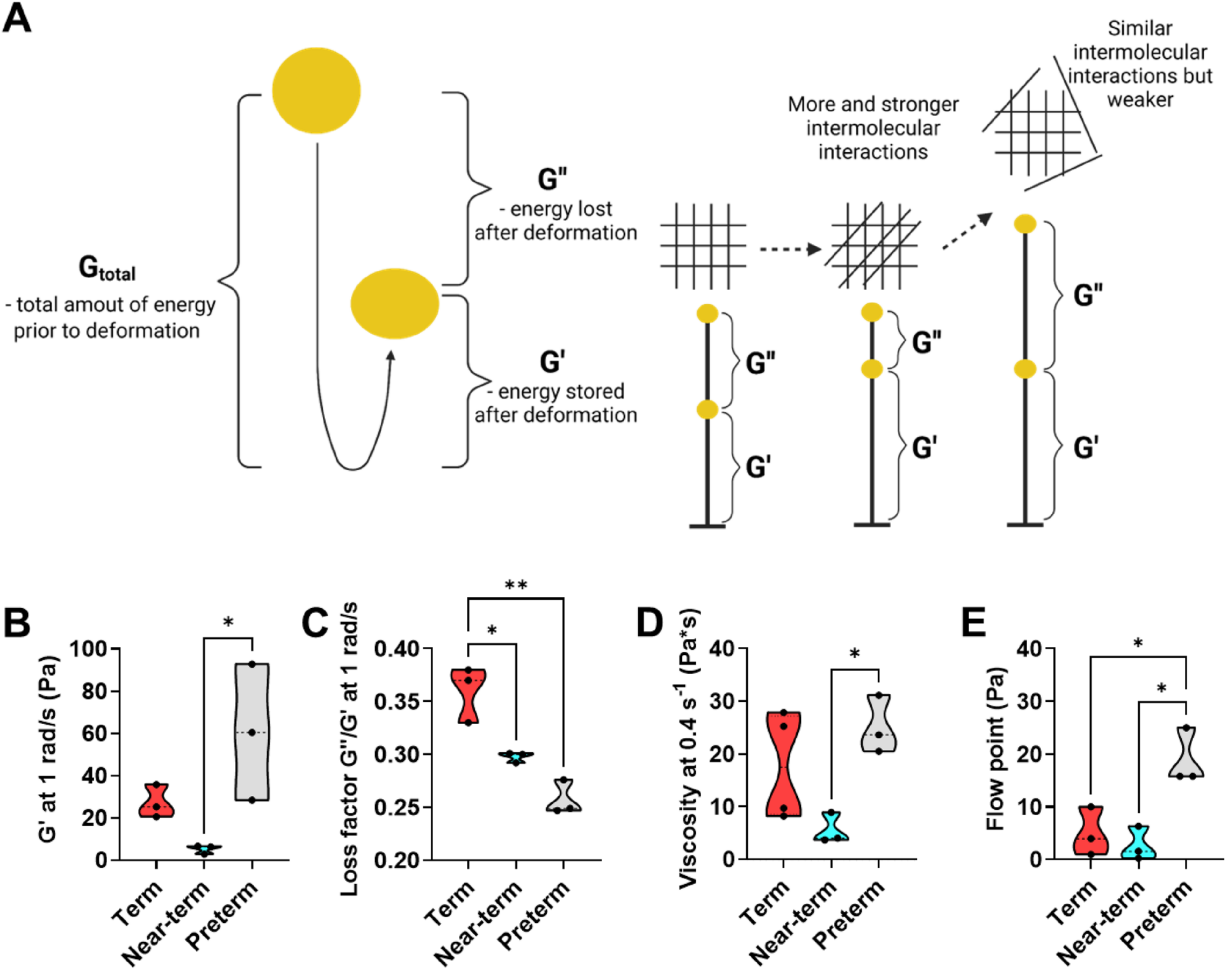
Rheological properties of ex vivo small intestinal mucus from 5-day old healthy piglets born with different birth maturities. (**A**) Schematic representation of the storage modulus (G′) and loss modulus (G″) obtained from small deformation rheology of mucus, and how this may relate to differences in the structure of mucus (illustration created with Biorender.com). (**B**) Elasticity i.e.,G′ and (**C**) loss factor at 1 rad/s of mucus obtained from the frequency sweep with a constant oscillation stress of 0.1 Pa. (**D**) Viscosity at 0.4 s^−1^ of mucus obtained with a continuous flow step. (**E**) Flow point (i.e., minimum oscillatory stress needed for G′ < G″ transition) of mucus obtained from a stress sweep with a constant frequency of 1 rad/s at a 0.001–500 Pa range (N = 3–4, n = 1). The data obtained from individual piglets are indicated by the black circles. For (**B**) and (**C**), one outlier was identified for the term group and was therefore not included in the presented data. Statistically significant differences are indicated as follows: *(*p* < 0.05) or **(*p* < 0.01). The exact *p* values are listed in the Supplementary Information Table S4. Source data are provided as a Source Data file.

The porous network of mucus goverens mucus´ bulk permeability as well as diffusitivity. Therefore, we next sought to examine whether the rheological properties of mucus were altered in piglets with different birth maturities. To this end, we conducted assessments of G' and G'' in the linear viscoelastic region of the collected mucus samples. As illustrated in Fig. 4A, G′ represents the amount of stored energy in the mucus network after deformation, whereas G″ represents the energy that has been lost after deformation. Hence, G′ can be related to the number of intermolecular interactions (i.e., elasticity) in the porous network of mucus, whereas the G″/G′ ratio (loss factor) represents the strength of these intermolecular interactions within the porous network of mucus^20^.

All mucus samples exhibited gel-like behavior (i.e., G′ > G″) in the 0.1–10 rad/s range (Supplementary Information Fig. S4A). Mucus from term and preterm piglets showed higher elasticity than mucus from near-term piglets, with G' being 5- and 11-fold higher, respectively, compared to near-term piglets (Fig. 4B). In contrast, the loss factor, G″/G′, appeared to increase with increasing birth maturity (Fig. 4C), driven by the differences in G″ (Supplementary Information Fig. S4B). This suggests that preterm birth (preterm vs. near-term) resulted in mucus with more and stronger intermolecular interactions, whereas cesarean section delivery and rearing with infant formula (near-term vs. term) resulted in mucus with fewer but stronger intermolecular interactions compared to mucus from vaginally delivered piglets.

Next, we measured the viscosity of mucus at increasing shear rates, as well as G′ and G″ at increasing oscillation stress to assess conditions mimicking the passage of food (Supplementary Information Fig. S5 and S6A). Similar to the G' data (Fig. 4B), mucus from preterm piglets exhibited a fivefold higher viscosity at a biologically relevant shear rate of 0.4 s^−1^^31^, as well as a sevenfold higher flow point (the minimum oscillation stress needed for G′ < G″ transition) compared to mucus from near-term piglets (Fig. 4D, E). Collectively, the rheological information illustrated in Fig. 4 and Fig. S6B indicated that there was an overall trend of near-term < term < preterm piglets, with the only exception being the ranking of the loss factor.

The combined data indicate that preterm birth (i.e., preterm vs. near-term) significantly affected both the barrier and rheological properties of mucus, whereas the delivery method and rearing (i.e., near-term vs. term) had less of an effect. However, it should be noted that the preterm, near-term and term piglets were raised on different diets, which may have affected the mucosal development. Though these dietary differences would in the clinical setting to cover the nutritional need of preterm, near-term and term infants. Intestinal mucus is known for its highly selective size filtration and interactive properties, which make passage through mucus highly dependent on the physicochemical properties of the permeating compound^20,28,32,33^. This may explain why mucus from preterm piglets exhibited both the slowest NP diffusion rates and the highest permeation of the small molecule and macromolecular model compounds compared to mucus from near-term and term piglets. These findings also indicate that mucus size filtration and interactive properties are dependent on birth maturity.

### Both perinatal asphyxia and necrotizing enterocolitis altered mucus properties

**Figure 5:**
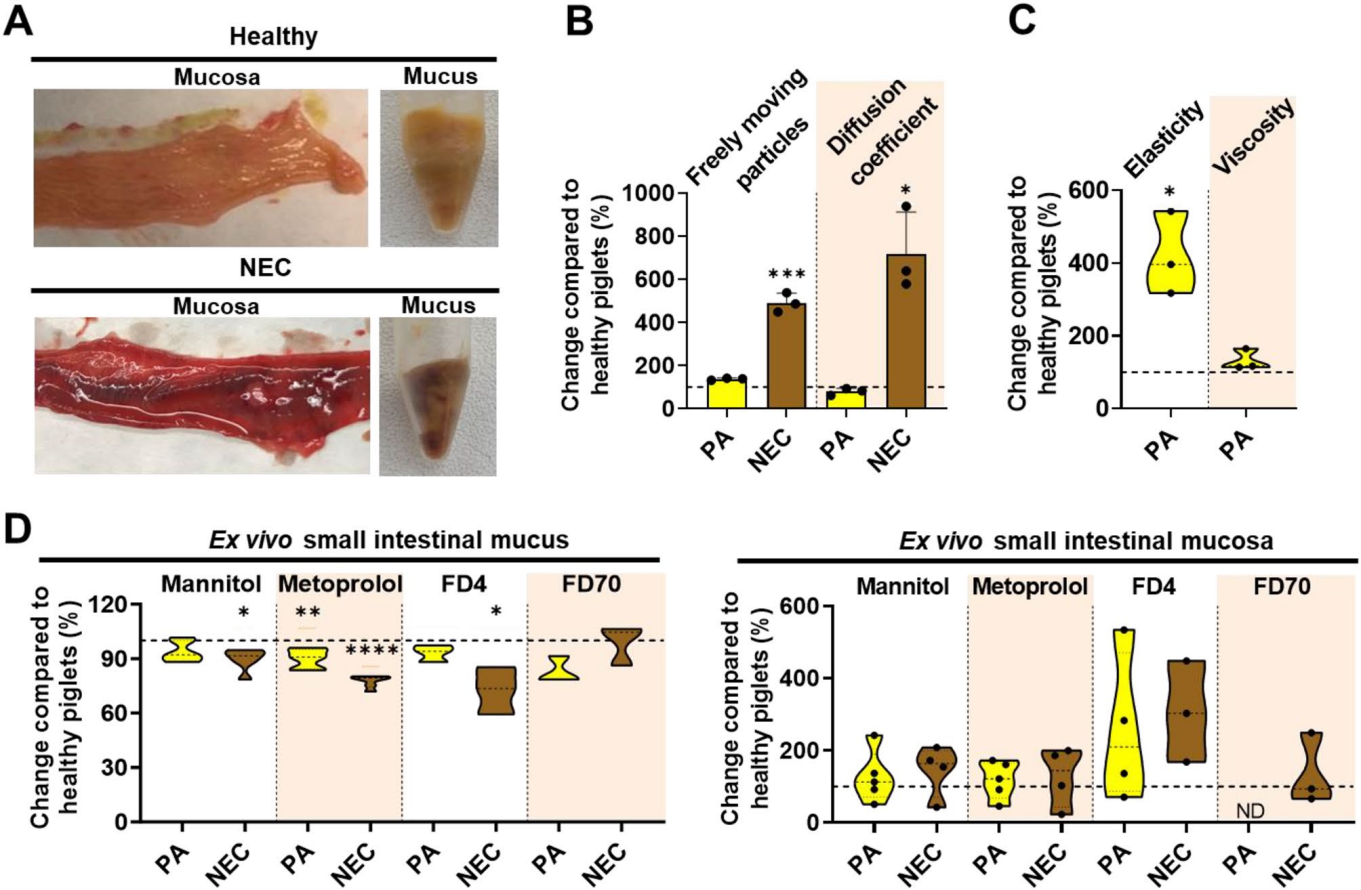
Effects of health conditions, perinatal asphyxia (PA) and necrotizing enterocolitis (NEC), on the rheological and barrier properties of ex vivo small intestinal mucus alone or the entire mucosa collected from piglets postnatal day 5. (**A**) Pictures of healthy and NEC mucosa and mucus. (**B**) Change in the fraction of freely moving nanoparticles and their diffusion coefficients in mucus from PA and NEC piglets compared to that in mucus from healthy piglets (N = 3, n = 2). (**C**) Change in storage modulus G′ (elasticity) at 1 rad/s and viscosity at 0.4 s^−1^ of mucus from PA and NEC piglets compared to that of mucus from healthy piglets (N = 3–4, n = 1). (**D**) Change in the apparent permeability coefficients (P_App_ values) of ^14^C-mannitol, ^3^H-metoprolol, fluorescein-isothiocyanate dextran 4 kDa (FD4), and fluorescein-isothiocyanate dextran 59–77 kDa (FD70) through ex vivo small intestinal mucus (N = 1, n = 3–6) or entire mucosa (N = 3–5, n = 1–2) from PA and NEC piglets compared to that of healthy piglets. Due to low sample volume, mucus from a minimum of three piglets from each group was pooled and mixed for the mucus permeability study, after which technical replicates were analyzed. For (**B**–**D**), the dotted line at 100% indicates the values for the healthy piglets. The variation in the number of piglets used for the mucosa permeability study was due to the study design and the exclusion of some data points that were below our lower limit of quantification. *ND* not detected. The black circles indicate data obtained from individual piglets. Data in the bar plots are presented as means with standard deviation. Significantly different data from that obtained from the healthy piglets are indicated as follows: *(*p* < 0.05), **(*p* < 0.01), or ***(*p* < 0.001). The exact *p* values are listed in Supplementary Information Table S4. Source data are provided as a Source Data file.

We next sought to investigate how the properties of mucus and mucosa are affected in piglets with impaired health conditions known to negatively affect postnatal gut development, namely PA and NEC^6,7,9^. In the case of near-term piglets with PA, we specifically sampled small intestines that were macroscopically evaluated as "healthy" (with a score of 1–2^34,35^) to examine whether the PA condition altered the mucus properties. This analysis aimed to provide insights into why infants with PA may have a higher risk of developing NEC^9,10^. For preterm piglets with NEC, impaired small intestines (with a macroscopic NEC score of 3–6^34,35^) were collected. Representative images of both healthy and NEC-impaired intestinal mucus and mucosa are shown in Fig. 5A.

The diffusitivity and bulk permeability of mucus as well as mucosa permeability in near-term piglets with PA were generally similar to that of healthy near-term piglets (Fig. 5B, D). Only the elasticity of mucus was increased by (418 ± 114%) in near-term piglets with PA (Fig. 5C).

**Figure 6:**
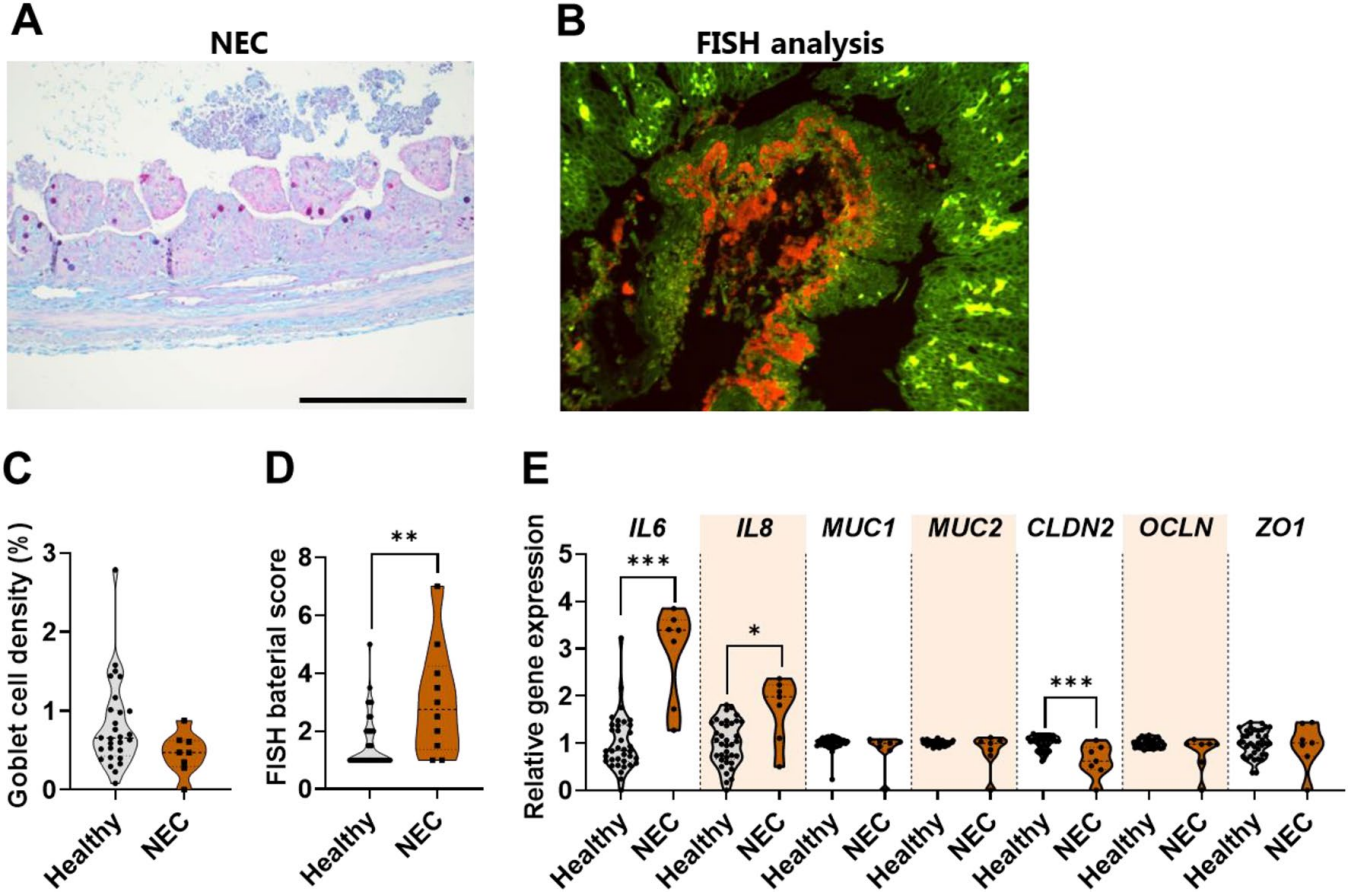
Effect of necrotizing enterocolitis (NEC) on goblet cell density, bacteria attachment, and expression of genes related to inflammation, mucin production, and selected tight junction proteins. (**A**) Representative micrographs of Alcian blue and periodic acid-Schiff (AB-PAS) stained section of the distal small intestine from piglets with NEC (scale bar: 500 µm). (**B**) Representative picture of FISH analysis of the distal small intestinal tissue with a red fluorescent-conjugated bacterial probe. (**C**) Goblet cell density (N = 8–28) and (**D**) FISH score of distal small intestine of healthy and NEC 5-days-preterm piglets (N = 7–37). (**E**) mRNA levels of target genes related to inflammation, mucin production, and tight junction representation in piglets with and without NEC (N = 7–37). The black circles indicate data obtained from individual piglets. Statistically significant data are indicated as follows: *(*p* < 0.05), **(*p* < 0.01), or ***(*p* < 0.001). Exact *p* values are listed in Supplementary Information Table S4. Source data are provided as a Source Data file.

Interestingly, for the NEC condition, the P_APP_ values of the smaller model compounds through mucus decreased with increasing molecular weight and hydrophobicity (mannitol < metoprolol < FD4) compared to healthy preterm piglets (Fig. 5D). In contrast, the diffusion coefficient and fraction of freely moving NPs were significantly increased by seven- and fivefold, respectively (Fig. 5B), in mucus from NEC preterm piglets. This suggests that mucus from NEC intestines may be significantly more prone to microbial penetration but less permeable to nutrient-sized compounds than mucus from healthy preterm piglets. Indeed, we found higher levels of bacteria adhering to the intestinal mucosa (Fig. 6B, D), as well as increased expression levels of innate inflammatory cytokines (*IL6*, *IL8*) (Fig. 6D, E) in NEC preterm piglets compared to healthy preterm piglets. Moreover, although the NEC preterm piglets tended to exhibit a visually disrupted mucus layer with fewer acidic mucins (stained blue) (Fig. 6A), no differences in the density of goblet cells or the gene expression level of the predominant gel-forming intestinal mucin, *MUC2*, were observed between the NEC and healthy preterm piglets (Fig. 6C, E). We observed downregulation of the gene coding for the tight junction protein claudin 2 (*CLDN2*) (Fig. 6E), which could explain the slightly more leaky mucosa (FD4 and F70) observed for the NEC preterm piglets (Fig. 5D). Collectively, our observations indicate that the NEC condition is associated with a compromised intestinal mucus barrier, which may facilitate microbial penetration through the mucus layer and, in consequence, inflammation of the intestinal barrier^1,2^.

### Health conditions and birth maturity affect sodium decanoate-induced alterations of mucus

Medium-chain fatty acids such as C10 have been reported to improve overall gut function when administered to infant mice and piglets, in addition to providing protection against cyclophosphamide-induced small intestinal dysfunction in pigs^18,19,36^. Moreover, the addition of C10, as well as another medium-chain fatty acid (sodium 8-[(2-hydroxybenzoyl)amino]octanoate), significantly increased the viscosity of ex vivo porcine intestinal mucus^37^, which we previously correlated with enhanced barrier properties of ex vivo porcine intestinal mucus^20^. Based on these findings, we investigated whether the addition of C10 would also improve the barrier properties of mucus from piglets suffering from PA and NEC.

**Figure 7:**
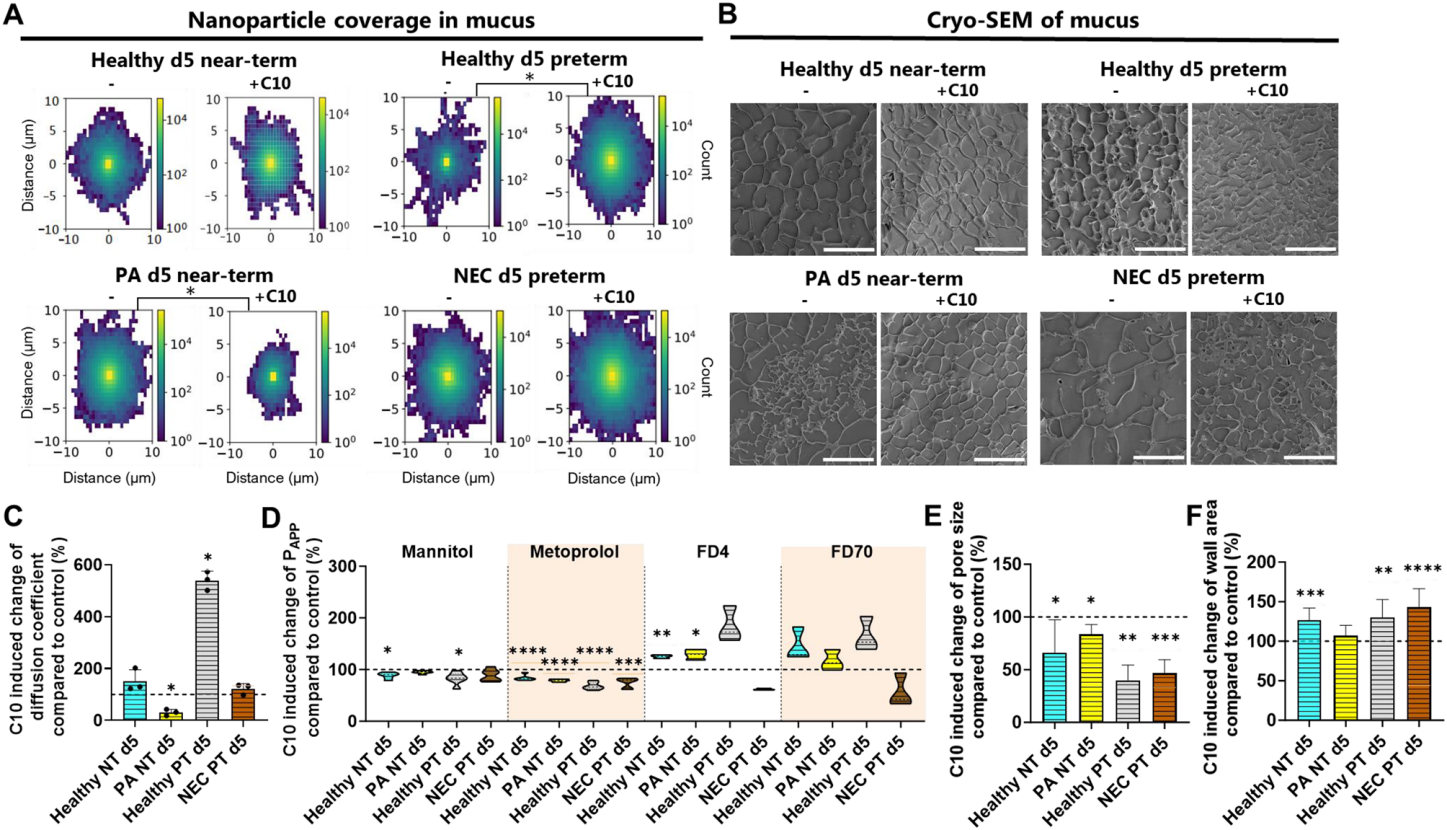
Effect of 25 mM sodium decanoate (C10) on physical and barrier properties of ex vivo small intestinal mucus collected on postnatal day 5 from either healthy, perinatal asphyxia (PA) or necrotizing enterocolitis (NEC) piglets with different birth maturities. (**A**) 2D heatmap of average (98% quantile) nanoparticle coverage in mucus in the presence and absence of C10 obtained using single particle tracking (N = 3, n = 2). (**B**) Representative cryo-scanning electron microscopy (cryo-SEM) images of mucus collected from healthy, PA, and NEC piglets in the presence and absence of C10 (Scalebar: 10 µm). C10 induced changes in (**C**) diffusion coefficients of nanoparticles in mucus (N = 3, n = 2) and (**D**) apparent permeability coefficients (P_App_ values) of ^14^C-mannitol, ^3^H-metoprolol, fluorescein-isothiocyanate dextran 4 kDa (FD4), and fluorescein-isothiocyanate dextran 59–77 kDa (FD70) through ex vivo small intestinal mucus (N = 1, n = 3–6) compared to that in the absence of C10 (dotted line). Due to low mucus volume, mucus from a minimum of three piglets from each group was pooled and mixed for the mucus permeability study, after which technical replicates were analyzed. Change in (**E**) pore size and (**F**) pore wall area in mucus calculated from the obtained cryo-SEM images in the presence of C10 compared to that in the absence of C10 (dotted line) (N = 1, n = 9–13). The black circles indicate data obtained from individual piglets. Data in the bar plots are presented as means with standard deviation. Significantly different data than obtained from mucus in absence of C10 are indicated as follows: *(*p* < 0.05), **(*p* < 0.01), ***(*p* < 0.001), or ****(*p* < 0.0001). The exact *p* values are listed in Supplementary Information Table S4. The birth maturities of the 5-day-old (d5) piglets were either near-term (NT) or preterm (PT). Source data are provided as a Source Data file.

The addition of 25 mM C10 to mucus from PA near-term piglets significantly decreased the NP coverage by 72 ± 14% and their diffusion coefficient by 70 ± 12% (Fig. 7A, C) compared to mucus alone. This was correlated with an increased confinement of NPs in mucus from PA near-term piglets (Supplementary Information Fig. S7) likely due to the tightened network in the mucus in the presence of C10 (Fig. 7B). Conversely, C10 did not affect the movement of NPs in mucus from the healthy near-term piglets, (Fig. 7A, C). This different C10-effect for PA and healthy near-term piglets was quite surprising, as NP movement in mucus in the absence of C10 was similar for PA and healthy near-term piglets (Fig. 5B). This suggests that mucus from the PA near-term piglets may have different interactive properties compared to mucus from healthy near-term piglets, and that C10 addition may minimize microbial penetration in mucus from the PA near-term piglets but not in healthy near-term piglets.

Interestingly, NP movement in mucus from NEC preterm piglets was not affected by the presence of C10 (Fig. 7A, C), whereas the bulk permeation of the different model compounds through mucus decreased by 11–44% when C10 was added (Fig. 7D). These findings were in contrast with the observed effects of C10 in mucus from healthy preterm piglets. Here, the addition of C10 significantly increased the NP coverage and their diffusion coefficients by 342% and 439% in mucus, respectively (Fig. 7A, C). Similar effects were observed for the bulk permeation of the peptide (FD4) and small pathogen model compounds (FD70) through the mucus, which also tended to increase by 65–84% in the presence of C10, whereas the permeation of the smaller molecules, mannitol, and metoprolol, decreased by 17–32% in healthy preterm piglets (Fig. 7D). These findings confirmed that the barrier properties of mucus, both in the absence and presence of C10, are highly influenced by the physiochemical properties, e.g., molecular sizes (small molecules vs. macromolecules vs. 100–300 nm NPs) and surface chemistry (hydrophilic FD vs. hydrophobic polystyrene NPs) of the permeant. Additionally, by analyzing multiple cryo-scanning electron microscope images of the porous network of mucus (Supplementary Information Fig. S8), we found that C10 addition decreased the pore sizes and increased the pore wall density of mucus at the micrometer scale in all piglets, albeit with less pronounced effects in the PA near-term piglets (Fig. 7E, F). This contrasted with the nanometer scale, where the NP diffusivity in mucus was increased for the healthy preterm piglets in presence of C10. Furthermore, we observed that the effect of C10 on NP diffusion in mucus was different for healthy near-term and preterm piglets.

Overall, the various effects observed with C10 in mucus indicate that the PA and NEC conditions, as well as the birth maturity of the piglets, may alter the interactive properties of mucus and that C10 has various effects at different length scales (e.g., micrometer-scale vs. nanometer-scale particles). It should be noted, that dietary difference between preterm and near-term piglet may have contributed to the observed differences in birth maturity and C10-effect. Though these diet differences would also have been presencent in the clinic.

## Discussion

During the prenatal period, the intestine develops in a protective sterile environment in utero, whereas the postnatal environment exposes the intestinal mucosa to numerous of opportunistic bacteria. The mucus barrier protects the underlying epithelium from bacterial invasion, and although the intestines of term-born infants are mature and ready to meet these demands, the immature intestines of preterm infants may struggle to keep out potential pathogens, resulting in 5–10% of preterm infants developing NEC^4,5,8^. Previous studies have shown a clear correlation between the risk of NEC with formula feeding and microbial dysbiosis^10,38–41^. However, the underlying mechanisms of NEC onset and the role of the mucus barrier in NEC development have not been fully revealed. By using surplus ex vivo intestinal mucus and mucosa samples, we discovered that the neonatal mucus barrier is highly complex in terms of its composition, physiochemical properties, and barrier function, which are dependent on postnatal age, birth maturity, and health conditions.

Here, we found that the intestinal mucus barrier changed significantly within the first few days of life, i.e., postnatal day 5 versus day 8/9, for both term and preterm piglets, but not in a similar manner. The barrier properties of the intestinal epithelium, including the mucus barrier, are generally considered to improve with increasing postnatal age for both term and preterm infants, as previously reported^15,33,42,43^, consistent with our findings for the term piglets. However, for preterm piglets, increasing postnatal age decreased the barrier properties of the intestinal mucus and the mucosa towards permeation of the large pathogen model compound (NPs) and macromolecules (FD4 and FD70), contradicting previous findings using standard in vivo gut permeability assays with lactulose and mannitol, i.e., small molecules^15,43^. The usage of small molecules are insufficient to evaluate whether larger compounds such as microbes are able to translocate across the intestinal mucosa^44^. Our findings suggest that the mucus barrier in preterm piglets are more complex and dynamic than previously thought. Despite the decreased mucus barrier properties postnatal day 8 for the preterm piglets, we found an increase in goblet cell density at this postnatal age. Intestinal goblet cells produce and secrete mucins, mainly MUC2, which are the primary structural components of the mucus layer in the intestine. Decreased goblet cell density is normally associated with decreased mucus thickness and increased mucus penetrability^3,14,33^, contradicting our findings. It is important to note that different litters of piglets were used for mucus collection and goblet cell determination in our study as well as the diets were different for the 5 days and 8/9 days preterm piglets. Large inter-litter variation could explain these observed differences in goblet cell density and mucus barrier properties at increasing postnatal age^45,46^. Yet, using the same piglet model, Ren et al.^15^ reported that distal small intestinal goblet cell density decreased with increasing postnatal age (day 1 vs. day 11) for preterm piglets but increased for term piglets, despite both term and preterm piglets had decreased gut permeability at day 11. Nevertheless, for the 5-day preterm piglets, we observed goblet cells containing mucins with both neutral and acidic PTMs, whereas 8/9-day preterm piglets with decreased mucus barrier properties displayed mainly neutrally charged PTMs. Alterations in the PTMs of mucin are known to be crucial for the overall barrier properties of mucus, as the specific PTM structures of MUC2 were found to be essential contributors to the mucus' ability to trap bacteria and prevent the onset of inflammatory bowel disease^22,23,47–49^. Additionally, in line with our observations, the presence of acidic mucins is generally believed to protect against bacterial translocation due to their higher stability toward bacterial degradation^22,33,49^. Hence, the PTMs of mucin may play a significant role in determining mucus barrier properties at different postnatal ages and health conditions.

Despite the relatively low goblet cell density in the 5-day preterm piglets, mucus from these preterm piglets was significantly more efficient in confining the NPs (large pathogen model compound) compared to mucus from term and near-term piglets. These findings were rather counterintuitive, as premature infants are generally considered to have poor intestinal barrier properties compared to term infants^4,15,43^. However, given that we first assessed the barrier properties of mucus at postnatal day 5 and NEC onset in this preterm piglet model is usually seen postnatally at day 2–4, it is possible that microbial stimuli before day 5 led to alterations in mucin structure and mucus composition, as reported in previous studies^3,33,50,51^. In turn, this could enhance the barrier properties of the mucus to minimize exposure of the premature intestinal epithelium to microbes. This hypothesis supports the increased confinement of our large pathogen model compound observed in mucus from preterm piglets, which coincides with more and stronger intermolecular interactions in the mucus, resulting in higher mucus viscosity compared to that of near-term and term piglets. Although our findings demonstrated that the barrier properties of mucus at postnatal day 5 are highly dependent on the birth maturity of the piglets, further studies are needed to understand if mucus composition, and therefore barrier properties, are dependent on gestation age (preterm vs. term infants) prior to microbial stimuli, or if our findings are solely due to the premature mucus barrier being affected by microbial stimuli. Additionally, it is important to note that different diets were used for the preterm, near-term and term piglet. Diet differences is well known to affect the intestinal development^46,52–54^, which may have contributed to the observed birth maturity differences. Though these diet differences would also have existed in the clinic.

In contrast to the healthy preterm piglets, the preterm piglets that developed NEC showed a different pattern. In these NEC piglets, the mucus barrier was unable to trap and confine the NPs (large pathogen model compound), which was consistent with higher levels of bacteria adhering to the NEC intestinal mucosa and increased expression levels of innate inflammatory cytokines (*IL6* and *IL8*), as previously reported^34,45^. However, we did not observe any significant differences in goblet cell density or gene expression of the predominantly intestinal gel-forming mucin, *MUC2*, in the NEC piglets. This suggests that the amount of mucins does not differ in the NEC condition and that other factors such as mucus composition and mucin PTM structure may regulate the mucus barrier instead. Although the NEC condition in our preterm piglets corresponds to a more sub-clinical NEC condition than in human infants^34^, we found that the mucus barrier was significantly impaired by this sub-clinical NEC state. This indicates that the mucus barrier may be an important factor in the NEC condition. However, additional studies are needed to identify the mechanisms through which the mucus barrier changes in response to NEC (*e.g*., mucus composition and PTM structure). The information gathered from such studies could be used for the identification of biomarkers for infants at risk of developing clinical NEC, in addition to providing a valuable basis for the medical community to better understand the pathogenesis of NEC and possible treatments.

In addition to NEC, we also studied the impact of PA on the barrier properties of mucus. Initially, the mucus barrier did not seem to be affected by the PA condition. However, significant differences in mucus barrier properties were observed upon C10 addition. These findings suggest that the interactive properties of mucus are altered with PA. Further studies are thus needed to understand whether this suggests that mucus from PA infants interacts differently with pathogenic microbes, which could potentially explain why infants with PA have a higher risk of NEC^9,10^.

Medium-chain fatty acids such as C10 are commonly found in infant formula, though their concentrations depend on the blend of fat sources used^16,17^. Of total saturated fatty acid content in infant formula, Mendonca et al.^17^ reported that the C10 content ranged between 0.4 and 13.8%, whereas the C10 content in human breast milk was 1.37 ± 0.7%. Moreover, the use of C10 has been reported to improve overall gut function, in addition to providing protection against cyclophosphamide-induced small intestinal dysfunction in piglets^18,19,36,55^. In this study, we investigated whether C10 could enhance mucus barrier properties, as we previously observed with another medium-chain fatty acid^20^, in addition to exploring its potential applicability as a therapeutic agent for impaired mucus barrier conditions. Interestingly, the C10 addition significantly reduced NP diffusion in mucus from PA near-term piglets but not in mucus from NEC preterm piglets. However, at the micrometer scale, similar to bacterial length scale^26^, C10 significantly reduced the pore sizes of mucus from both healthy, PA, and NEC piglets, suggesting that C10 might reduce bacterial penetration in mucus by enhancing the micro-structure of mucus. Hence, C10 may be used to improve the barrier properties of mucus in infants. Nevertheless, the effects of C10 in mucus appear to be highly dependent on the health condition and birth maturity of the piglets, as well as the model compound assessed and the length scale in mucus at which the C10 effects were investigated. It should also be noted that the 5 days preterm and 5 days near-term received different infant formulas, which may have contributed to the characteristics of mucus. Furthermore, although C10 is approved as a food additive^56^, used in infant formula^17^, and tested at the gram scale without any reported genotoxicity or subchronic toxicity^57^, this compound is also known to increase transmucosal permeation of macromolecules when used within a two- to three-digit mM range in rodents in vivo^58,59^. Nevertheless, further work is needed to understand how C10 interacts with mucus and explore any opportunities to improve conditions with impaired mucus barrier.

In conclusion, paying attention to the complexity of mucus from infants is an important step towards understanding how the mucus barrier develops postnatally and how it may play vital roles in disease conditions. Our novel use of advanced complementary techniques provides important insights into the postnatal variations of the mucus barrier properties and shows that size filtration and interactive properties of neonatal mucus are highly dependent on postnatal age, birth maturity, and health condition. Collectively, our findings highlight the role of neonatal mucus as an essential and highly regulated barrier, as well as its potential involvement in disease onset. However, additional studies are needed to understand how the functions of the neonatal mucus barrier are governed by mucin PTM structures and mucus composition, as well as how these processes are regulated prior to and during disease onset.

## Methods

### Materials

Fluorescein-isothiocyanate dextran of 4 kDa (FD4) and 59–77 kDa (FD70), Alcian blue 8Gx, and Hank's Balanced Salt Solution (HBSS) were purchased from Sigma-Aldrich (Søborg, Denmark). Sodium decanoate > 99% (C10) and MES anhydrous BioChemica (MES) were acquired from Tokyo Chemical Industry Co. (Tokyo, Japan) and PanReac AppliChem (Darmstadt, Germany), respectively. Sucrose was bought from WVR (Søborg, Denmark), and Ringer tablets, periodic acid solution, and Schiff's reagent were obtained from Merck KGaA (Darmstadt, Germany). SPHERO™ Fluorescent Nile Red Particles with a diameter of 0.1–0.3 µm were purchased from Nordic BioSite (Copenhagen, Denmark). Mannitol D-[1-^14^C] (57.2 mCi/mmol) and Ultima Gold scintillation fluid were purchased from Perkin Elmer (Ballerup, Denmark). Metoprolol-[^3^H] (27.6 Ci/mmol) was obtained from Vitrax Radiochemicals (Placentia, CA, USA). Alexa Fluor 555-conjugated oligonucleotides targeting bacterial 16S RNA (sequence: 5′-GCTGCCTCCCGTAGGAGT-3′) were synthesized by Eurofins Genomics (Ebersberg, Germany). Primers for intestinal gene expression were designed based on published sequences of target genes in pigs using the NCBI online primer design tool (Primer-BLAST: http://www.ncbi.nlm.nih.gov/tools/primer-blast/). Freshly prepared ultrapure water (18.2 MΩ × cm) purified by a PURELAB flex 4 (ELGA High Wycombe, UK) was used if not otherwise stated.

### Ethics and experimental design

All experimental procedures were conducted in accordance with the European Union Directive 2010/63 for the use of animals in research and were approved by the Danish Animal Experiments Inspectorate (License No. 2020-15-0201-00,520), and reported in accordance with ARRIVE guidelines. The study used crossbred piglets (Landrace × Yorkshire × Duroc), delivered by cesarean section at gestational day 106 (90%, preterm) or 113 (97%, near-term), or delivered vaginally at day 117 (term). Preterm and near-term piglets were reared individually in incubators or cages and fed with formula based on bovine milk. The 5 days preterm piglets were fed using the formula PreNan Preemie from Nestle, whereas the other preterm and near-term piglets were fed with a mixture of whey protein concentrate from Arla Foods, Liquigen/Calogen (lipid) and SHS Seravit (vitamin and minerals) from Nutricia with composition and energy adjusted for nutritional requirement for newborn piglets^54^. The piglets were reared for 5, 8–9, or 19 days, depending on the experimental group. Term piglets were delivered and reared at a Danish commercial farm until the piglets were euthanized for tissue collection by pentobarbital via an intercardiac overdose. For anasthesia, a combination of zolazepamtiletamin(Zoletil 50; Virbac, Kolding, Denmark), xylazin (20 mg/ml, Narcoxyl;MSD Animal Health, Ballerup, Denmark), ketamine (100 mg/ml,Ketaminol; MSD Animal Health, Rahway, NJ, USA), and butorphanol (10 mg/ml, Torbugesic; ScanVet, Fredensborg, Denmark) was administered via intramuscular injection. In the near-term piglet group, half of the litter was exposed to 6 min of cord occlusion before delivery to induce fetal hypoxia, mimicking birth asphyxia commonly occurring in preterm and near-term infants (perinatal asphyxia, PA). The NEC condition was spontaneously induced in our well-established preterm piglet model by feeding the piglets with infant formula. In this model, 33% of the piglets in the sampled litters developed NEC in the small intestines. Piglets were categorized as either healthy (score of 1–2) or NEC (score of 3–6) based on macroscopic evaluation of different regions of the gastrointestinal tract using our well-established NEC scoring system^34,60^. For the 5-day preterm piglets, the NEC finding was solely based on the score of the small intestine.

### Isolation of piglet intestinal tissues and mucus

After euthanasia, various segments of the gastrointestinal tract, including the stomach, proximal, middle, and distal small intestine, and colon, were dissected for pathology evaluation and collection of fresh tissue. Distal small intestinal tissue used for ex vivo mucosa permeability studies was kept in ice-cold Ringer's solution (pH 7) with 10 mM sucrose (sRinger’s) for up to 12 h prior to the studies. Formalin-fixed distal small intestinal tissues were used for staining and quantification of goblet cell density and fluorescence in situ hybridization (FISH) analysis. Snap-frozen middle small intestinal tissue was used for gene expression analysis via real-time quantitative polymerase chain reaction (RT-qPCR). Mucus was collected from the remaining small intestine by gently scraping the mucosal surface and used for single particle tracking, rheological profiling, and cryo-scanning electron microscopy (cryo-SEM). The piglets were not fasted prior to euthanasia, and therefore the collected mucus contained some food residues.

### Ex vivo intestinal mucosa permeability

To evaluate the impact of postnatal age, birth maturity, and health condition of the piglets, as well as C10 supplementation, isolated distal small intestinal tissue was mounted on Franz diffusion cells (0.9 cm in diameter, PermeGear, Hellertown, PA, USA) filled with sRinger’s, pre-heated to 37 °C using a heating rack (PermeGear) attached to a water bath (Julabo GmbH, Seelbach, Germany). Next, the test samples (250 µL) of 25 mg/mL FD4 with 4 µCi/mL ^3^H-metoprolol and 4 µCi/mL ^14^C-mannitol, or 25 mg/mL FD70 with 4 µCi/mL ^3^H-metoprolol and 4 µCi/mL ^14^C-mannitol, with or without 25 mM C10, were added. 200-µL samples were then collected from the receiver chamber every 30 min post-administration for 4 h, and the receiver volume was supplemented with 200 µL Ringer's solution at 37 °C. From the collected sample, 100 µL was transferred to a scintillation vial, and 2 mL scintillation fluid was added for quantification of ^14^C-mannitol and ^3^H-metoprolol using a Tri-Carb 2910 TR Scintillation analyzer (Perkin Elmer, Boston, MA, USA). For evaluation of FD4 and FD70, 100 µL of the collected samples were transferred to a Nunc 96-well microplate (Thermo Fischer Scientific, West Palm Beach, FL, USA). The fluorescence samples were analyzed using a plate reader (FLUOstar Omega, BMG LABTECH, Ortenberg, Germany) at λ_ex_ 485 nm and λ_em_ 520 nm. Measurements were conducted on a minimum of three different piglets with one replicate (N ≥ 3, n = 1). The apparent permeability coefficient (P_APP_) was calculated as previously described^20^.

### Permeability studies through bulk mucus

To study the permeability of bulk mucus and the effect of birth maturity and, the health condition of the piglets as well as C10 supplementation, 75 µL of freshly collected mucus was applied onto a Corning Transwell® insert (6.5 mm diameter polycarbonate membrane with 0.4 µm pore size; Sigma-Aldrich, Søborg, Denmark) and allowed to equilibrate for 10 min on a horizontal shaking table (50 rpm and 37 °C) (Thermo MaxQ 2000, Thermo Fisher Scientific, West Palm Beach, FL, USA). Afterward, 600 µL of 10 mM MES in HBSS pH 6.5 (mHBSS) was added to the receiver compartment and allowed to equilibrate with horizontal shaking for another 10 min. Next, 30 µL of test solutions consisting of either 250 µg/mL FD4 with 0.5 µCi/mL ^3^H-metoprolol and 0.5 µCi/mL ^14^C-mannitol, or 1000 µg/mL FD70 with 0.5 µCi/mL ^3^H-metoprolol and 0.5 µCi/mL ^3^H-mannitol, with and without C10 at a final concentration of 25 mM in the apical compartment, were applied. At 15–30 min intervals for 4 h, 200-µL samples were collected from the receiver compartment and replenished with 200 µL mHBSS (37 °C). Quantification and calculation of permeated ^3^H-metoprolol/^14^C-mannitol, FD4, and FD70 were conducted as described in the ex vivo intestinal mucosa permeability section. Measurements were conducted using pooled mucus from a minimum of 3 different piglets from each group due to low sample volume (N = 1, n ≥ 3).

### Rheological profiling

Rheological measurements of freshly collected mucus alone and in the presence of 25 mM C10 after 1 h of incubation at 37 °C were conducted using an AR-G2 rheometer (TA Instruments-Waters, New Castle, DE, USA) equipped with a truncated 1° 40 mm cone and a Peltier plate maintained at 37 °C. Sample dehydration was minimized using a solvent trap (TA Instruments-Waters, New Castle, DE, USA). The measurements were performed in three consecutive steps: (1) a frequency sweep in the range of 0.1–10 rad/s using an oscillation stress of 0.1 Pa, which is well within the linear viscoelastic range, as determined from the stress sweep (Supplementary Information Fig. S6A); (2) a continuous flow ramp step with shear rate of 0.001–3000 s^−1^; and lastly (3) a stress sweep with a constant frequency of 1 rad/s at an oscillation stress range of 0.001–500 Pa. The samples were allowed to equilibrate for 5 min between each measurement. Measurements were conducted on a minimum of three different piglets with one replicate (N ≥ 3, n = 1).

Evaluation of the rheological data was carried out by comparison of the storage modulus (G′) and the loss factor (ratio between loss modulus and storage modulus (G′/G″)) at 1 rad/s from the frequency sweep as well as viscosities at a biologically relevant shear rate, i.e.,0.4 s^−1^^28^, from the continuous flow ramp. Furthermore, assessment of the stress stability of the mucus, i.e., yeild point (the oscillation stress need to decrease G′ 5% from its stress independent region) and flow point (minimum oscillation stress needed for a G′ < G″ transition) were obtained from the stress sweep.

### Single particle tracking in mucus

To investigate whether nanoparticle diffusion in mucus is influenced by the postnatal age, birth maturity, health condition of the piglets, and C10 supplementation, 100–300 nm Nile Red labeled nanoparticles (NP) were tracked within the mucus. For single particle tracking (SPT), mucus samples were prepared by mixing freshly collected mucus with mHBSS alone or containing C10 and then incubated for 1 h at 37 °C. After incubation, the NPs were added to the mucus and vortexed to ensure that they were evenly dispersed within the sample. The mucus samples had a total hydration of 14.6% (v/v) with mHBSS and a final concentration of 0.08 mg/mL NP with either 0 or 25 mM C10. Following the preparation of the samples, a volume of 30 µL was added to a custom-made microscope chamber (aluminum with Teflon support) and imaged using an inverted spinning disk confocal microscope (Olympus SpinSR10, Olympus, Tokyo, Japan). The observations were conducted using a 60 × oil immersion objective with a numerical aperture of 1.4 (Olympus) and a CMOS camera (PRIME 95B, Teledyne Photometrics, Tucson, AZ, USA), which provided a final pixel dimension of 0.183 × 0.183 µm. Time series were recorded at 4.56 Hz, corresponding to a frame rate of 218 ms, using 30 ms exposure times and 532 nm laser excitation, resulting in a total of 300 frames per movie. NP identification, signal extraction, and background correction were conducted as described in our recent studies^20,61–63^. The images were obtained at room temperature in the x–y plane, with a ZDC current offset used to confine the recording in the z-axis. Measurements were performed on mucus samples from three different piglets, and two movies were collected at different positions in each mucus sample (N = 3, n = 2).

For data analysis and formation of trajectories, we used Python 3.8 × as described in a previous study^20^. The data from each biological sample were pooled and formed the basis for subsequent analysis of NP diffusion. To avoid ensemble biasing and thus provide a detailed view of all behaviors, the diffusion coefficient, mean square displacement and the anomalous diffusion parameter (alpha, α) were determined for each individual NP. The code used for analysis is provided as a Source Data file. To assess the confinement of the NPs, the alpha values were classified into three categories: immobile (α < 0.001), confined (0.001 < α < 0.5), and freely moving NP (α > 0.5). Freely moving NPs would display Brownian motion and an alpha value of 1, whereas a value of less than 1 would indicate a confined motion^63^. However, we previously demonstrated that when these NPs move in buffer (i.e., freely), they have an alpha value greater than 0.5, as they were traced only in the x–y dimension^20^. Furthermore, we visualized the spread of the NP from their original positions by arbitrarily adjusting all NPs to originate from the same point and allowing them to move out from that point. The resulting positions were compiled to form a 2D heatmap for qualitative comparison of the average NP coverage.

### Cryo-scanning electron microscopy (cryo-SEM)

For cryo-SEM, mucus alone or mucus incubated for 1 h with 25 mM C10 at room temperature was sandwiched between two 100 µm cavity planchettes and cryopreserved using liquid N_2_, as previously described^20^. The samples were then cracked, subjected to 4 min of sublimation at − 90 °C, and sputter coated with a 6 nm thick carbon/platinum layer using a Leica EM MED020 coating system (Leica, Vienna, Austria). Images were obtained using a Quanta 3D FEG SEM (FEI, Eindhoven, The Netherlands) with an accelerating voltage of 2 kV and a cryostage temperature of − 140 °C.

Images with a 10,000 magnification were used for further image analysis in MATLAB® R2021b (MathWorks, Natick, MA, USA). The images were initially cropped to remove the bottom footer. Image smoothing was performed by passing the images through a Gauss filter with a filter size of 10. The pore size was calculated by converting the pixels that constitute the pores into the equivalent µm^2^ area based on the utilized image zoom objective. The wall density was calculated by summing up the gradient magnitude in the image. The code is provided as a Source Data file. Images were obtained in samples from 1 piglet, and image analysis was conducted on a minimum of 8 representative images (N = 1, n ≥ 8).

### Staining of bacteria in intestinal mucosa by fluorescence in situ hybridization (FISH) and mucin-containing goblet cells

Formalin-fixed distal small intestinal tissues were used for quantification of bacterial abundance adhering in the mucosa via FISH analysis^64^. Briefly, paraffin-embedded 3-µm cross sections were stained with a general probe targeting bacterial 16S rRNA and visualized by fluorescence microscopy [AxioCam MRm version 3 FireWire monochrome camera with AxioVision version 4.5 (Carl Zeiss, Oberkochen, Germany)]. Fluorescence signals were blindly scored from 0 to 6, according to the bacterial density: 0, no bacteria; 1, very few bacteria; 2, few spread bacteria; 3, bacteria spread across the whole section; 4, small bacteria colonies across the section; 5, large colonies; and 6, overgrowth of large colonies (N ≥ 10, n = 1).

Another fixed distal small intestinal tissue was stained with Alcian blue (AB) and periodic acid-Shiff (PAS) for density quantification of mucin-containing goblet cells (N ≥ 28, n = 1) as previously described^65^. Depending on the color of the stained mucins in the goblet cells, the PTMs of the synthesized mucins can be elucidated, as neutral mucins are stained magenta by PAS, whereas only acidic mucins (i.e. sulfur and carboxyl PTM) are stained blue by AB^21,65^.

### Small intestine gene expression analysis

Gene expression analysis using RT-qPCR for targets related to inflammation, mucin production, and epithelium tight junction barrier was performed in the middle small intestinal tissues as previously reported^66^. The target genes included *IL6, IL8, MUC1, MUC2, CLDN2, OCLN,* and *ZO1*, in addition to the reference genes *B2M* and *SDHA.* The primer sequences (Supplementary Information Table S3) and procedures for relative expression measurements were the same as in previous studies^66–68^.

### Statistical analysis

Statistical analyses of the permeability of small intestinal mucosa and mucus, SPT, rheology, and image analysis of mucus cryo-SEM images were conducted using GraphPad Prism 9 for Windows (GraphPad Software, La Jolla, CA, USA). For mucosa permeability and rheology data containing more than 3 biological replicates, ROUT outlier analysis was performed with a Q of 5%, to account for possible mucosa integrity issues and presence of large solid particles in mucus, which would affect the mucosa permeability and rheology data, respectively. One-way analysis of variance (ANOVA) followed by Tukey's multiple comparison tests were conducted for assays with three or more groups, whereas pairwise comparisons were conducted via Student’s *t* test. Tests were paired when necessary, and in case of unequal variance, a Welch test was performed instead. For biological data, including FISH score, gene expression, and goblet cell density, statistical analysis was conducted using the JMP 14.0 software (SAS Institute, Cary, NC, USA). FISH scores comparing samples from NEC versus no NEC piglets were analyzed using the non-parametric Mann–Whitney test. FISH scores and distal small intestinal NEC scores were analyzed for correlation using Spearman's rank test. Gene expression and goblet cell density data were analyzed using a linear mixed model with time or NEC as a fixed factor and litter as a random factor. When necessary, log or square root data transformation was performed to ensure normal distribution. For all statistical tests, *P* values < 0.05 were considered statistically significant. All data are presented as means with standard deviations, with the number of biological replicates (i.e., number of piglets) indicated by N, whereas n indicates the number of technical replicates.

### Supplementary Information

Supplementary Information 1.

Supplementary Information 2.

## Data Availability

All data are available either in the article, the Supplementary data files, or from the corresponding authors upon reasonable request. The code used for calculation of wall density for cryo-SEM images is provided as a Source Data file.
