# Supplementary material for: Neonatal intestinal mucus barrier changes in response to maturity, inflammation, and sodium decanoate supplementation
Source: Sci Rep. 2024 Apr 1;14:7665. doi: 10.1038/s41598-024-58356-5 (PMC10985073; doi:10.1038/s41598-024-58356-5)
Supplement: Supplementary file 1 — Supplementary Information 1. [file 41598_2024_58356_MOESM1_ESM.docx]

**Supplementary Information for**

**Neonatal intestinal mucus barrier changes in response to maturity, inflammation, and sodium decanoate supplementation**

**Authors:**

Janni Støvring Mortensen^a^, Søren S.-R. Bohr^a,b^, Lasse Skjoldborg Krog^a^ , Johan Peter Bøtker^c^, Vaya Kapousidou^b^, Lasse Saaby^c,d^, Nikos Hatzakis^b,e^, Hanne Mørck Nielsen^a^, Duc Ninh Nguyen^f,^* & Stine Rønholt^a,^*

^a^ Center for Biopharmaceuticals and Biobarriers in Drug Delivery (BioDelivery), Department of Pharmacy, Faculty of Health and Medical Sciences, University of Copenhagen, Universitetsparken 2, DK-2100 Copenhagen, Denmark

^b^ Department of Chemistry and Nanoscience Center, Faculty of Science, University of Copenhagen, Universitetsparken 5, DK-2100 Copenhagen, Denmark

^c^ Department of Pharmacy, Faculty of Health and Medical Sciences, University of Copenhagen, Universitetsparken 2, DK-2100 Copenhagen, Denmark

^d^ Bioneer A/S, Kogle Alle 2, DK-2970 Hørsholm, Denmark

^e^ NovoNordisk Center for Protein Research, Faculty of Health and Medical Sciences, Blegdamsvej 3b, DK-2200 Copenhagen, University of Copenhagen Denmark.

^f^ Department of Veterinary and Animal Sciences, University of Copenhagen, Dyrlægevej 68, DK-1870 Frederiksberg C, Denmark

* Corresponding authors: [dnn@sund.ku.dk](mailto:dnn@sund.ku.dk) (D.N.N.) and stine.roenholt@sund.ku.dk (S.R.).


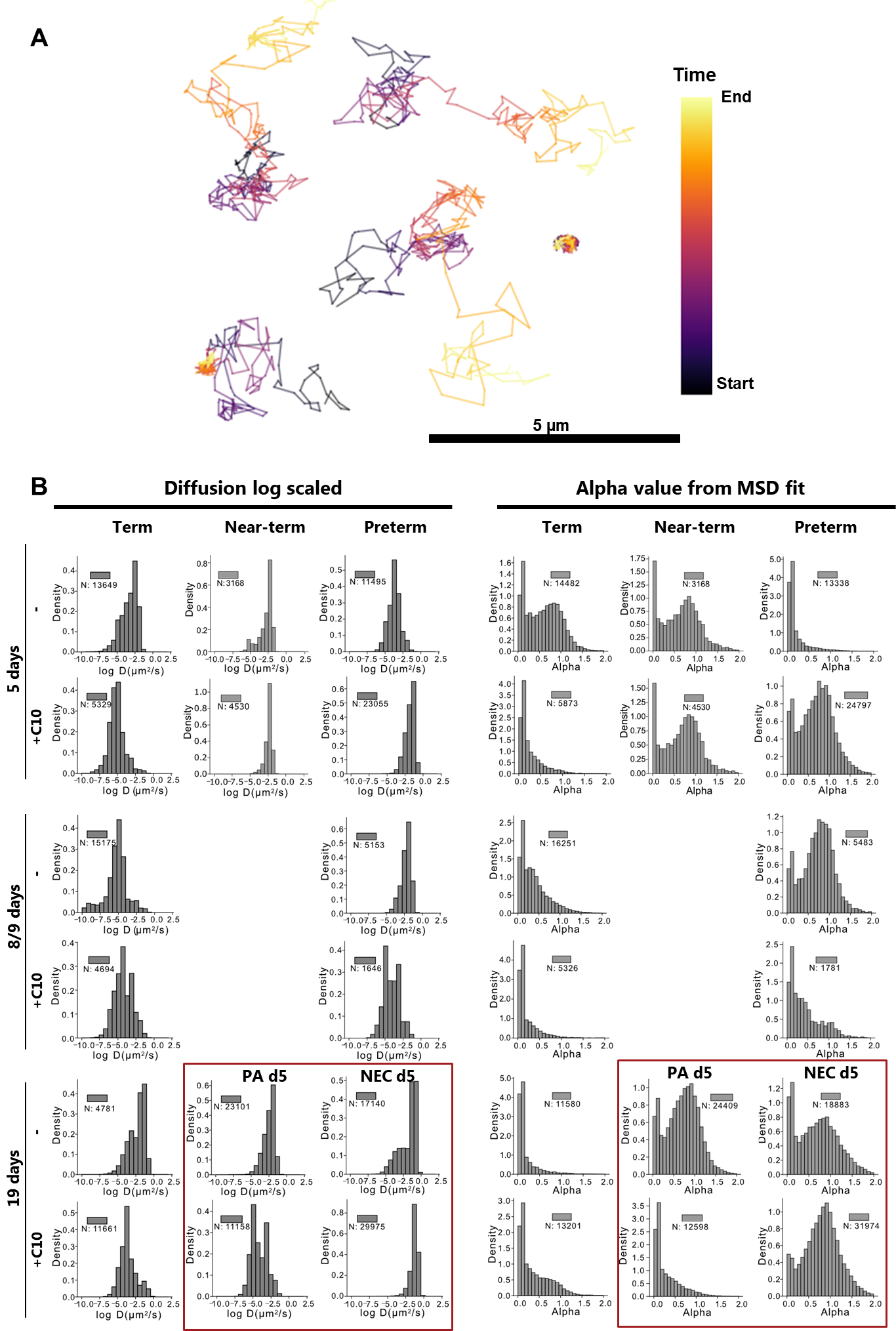


**Fig. S1.** **Nanoparticle tracking in *ex vivo* small intestinal mucus of from piglets in absence and presence of 25 mM sodium decanoate (C10).** Single particle tracking of 100–300 nm nile red polystyrene particles in intestinal mucus was obtained. Mucus was collected from 5–19 days old healthy, perinatal asphyxia (PA) or necrotizing enterocolitis (NEC) piglets that either were born term (gestational day 117) or delivered near-term (gestational day 113) or preterm (gestational day 106). Postnatal age of piglets is given in days *e.g.* d5 = 5 days old. **A)** Examples of tracks as function of time of five individual nanoparticles in mucus. **B)** Distribution of calculated instantaneous diffusion coefficients and anomalous diffusion parameter (alpha value), describing particle confinement, of the nanoparticles in mucus for each condition. For each condition data were pooled from N=3, n=2 and the number particles fitted are shown in each graph (trajectories where the fit did not converge has been removed). The diffusion coefficients were obtained using an unbinned maximum likelihood fitting of the individual trajectories, while the alpha value was obtain from the mean square displacement, explaining the differences in the number of particles fitted for the diffusion coefficient and alpha value for the same condition.

**
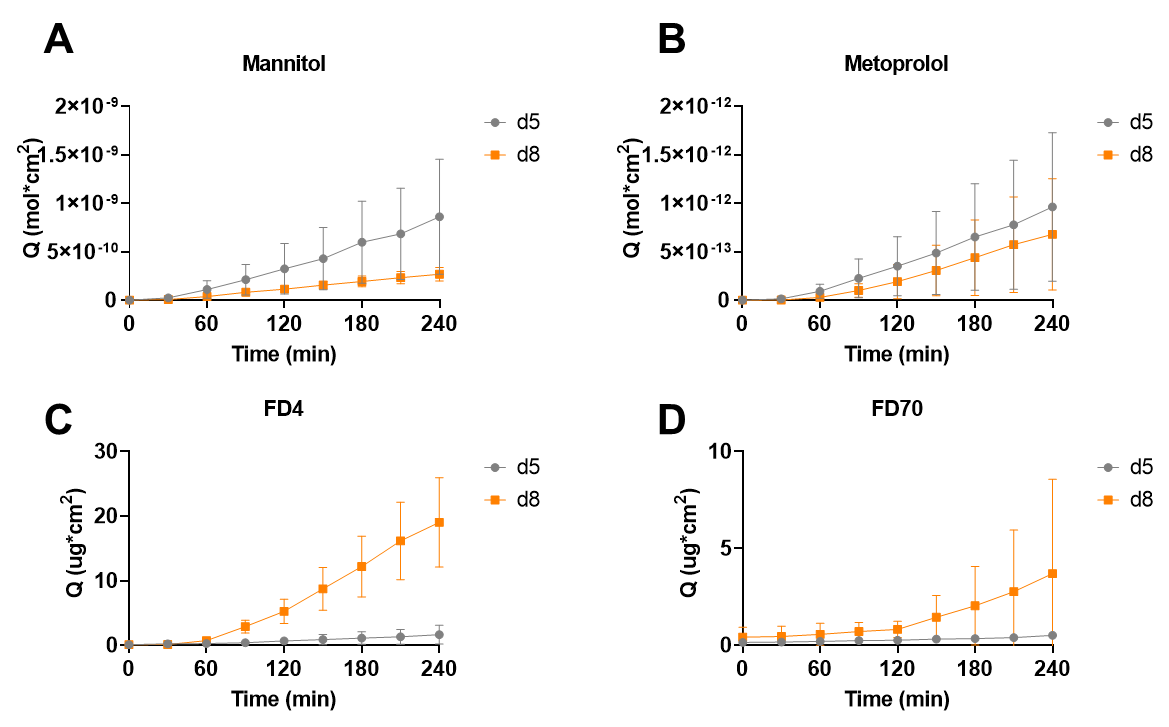
**

**Fig. S2**. **Effect of postnatal age on *ex vivo* small intestinal mucosa permeability from healthy preterm piglets.** Flux curves of model compounds´ permeation through *ex vivo* small intestinal mucosa of from 5 and 8 days old healthy preterm piglets. The permeation of **A)** ^14^C-mannitol, **B)** ^3^H-metoprolol, **C)** fluorescein-isothiocyanate dextran 4 kDa (FD4) and **D)** fluorescein-isothiocyanate dextran 59–77 kDa (FD70) through freshly isolated mucosa over time (N=3–6, n=1–2). The differences in y-axis levels for **A-D** were due to different test concentrations of ^14^C-mannitol (70 µM), ^3^H-metoprolol (0.15 µM), FD4 (25 mg/mL) and FD70 (25 mg/mL) that were applied to the mucosa. Data are presented as means with standard deviations. Outlier analysis was not performed on the depicted data. Postnatal age of piglets is given in days *e.g.* d5 = 5 days old. Source data are provided as a Source Data file.


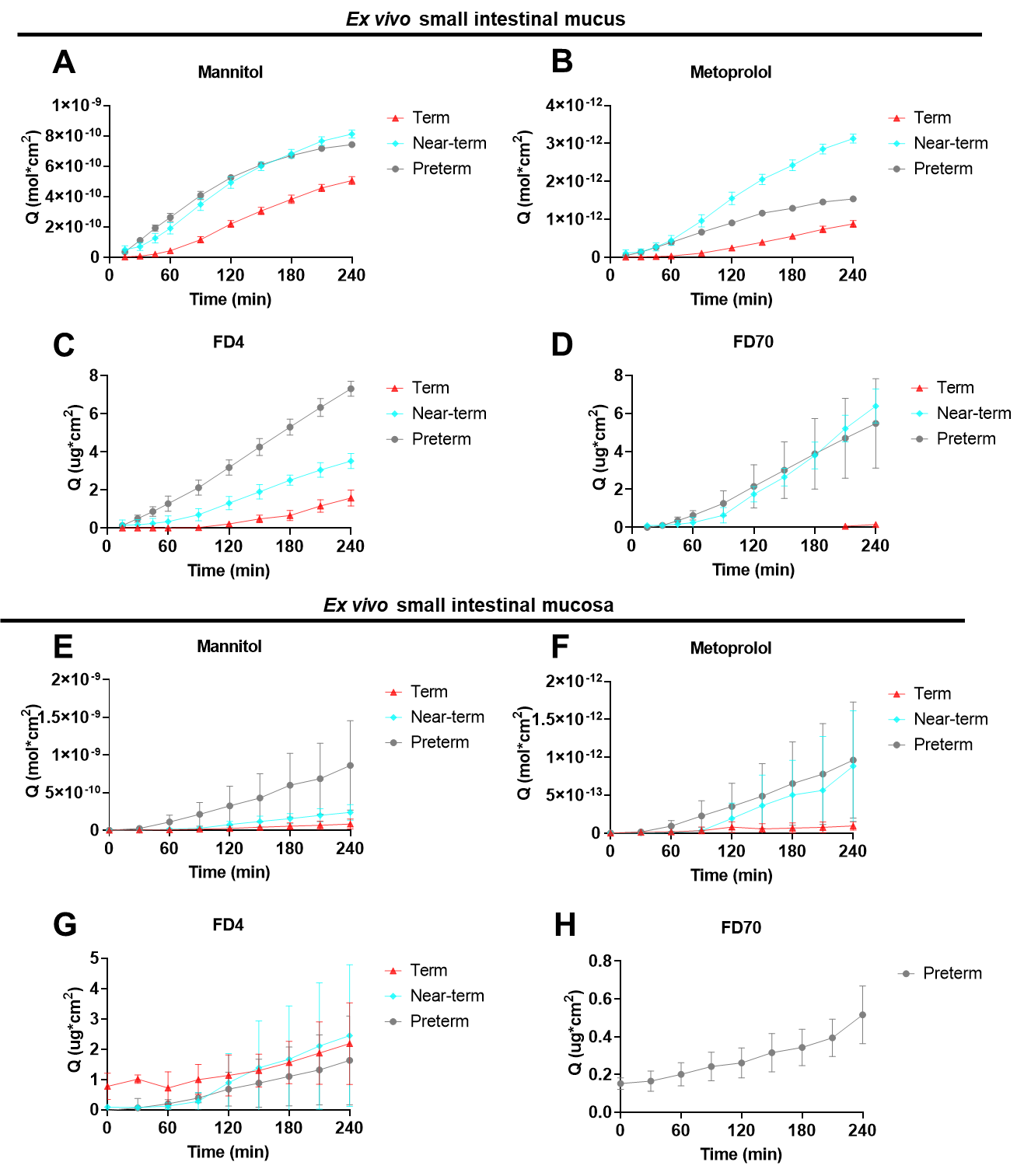


**Fig. S3**. **Effect of birth maturity of healthy piglets on *ex vivo* small intestinal mucus and mucosa permeability.** Flux curves of model compounds´ permeation through *ex vivo* small intestinal mucus and mucosa of from 5 day old healthy term, near-term and preterm piglets**.** The permeation of **A, E)** ^14^C-mannitol, **B, F)** ^3^H-metoprolol, **C, G)** fluorescein-isothiocyanate dextran 4 kDa (FD4) and **D, H)** fluorescein-isothiocyanate dextran 59–77 kDa (FD70) through freshly collected **A-D)** mucus (N=1, n=3–6) or **E-H)** mucosa (N=3–6, n=1–2) over time. The differences in y-axis level for **A-D** and **E-H** were due to different test concentrations of ^14^C-mannitol (8.74 and 70 µM), ^3^H-metoprolol (0.018 and 0.15 µM), FD4 (0.25 and 25 mg/mL) and FD70 (1.0 and 25 mg/mL) were applied to the mucus and mucosa, respectively. Data are presented as means with standard deviations. Outlier analysis was not performed on the data depicted. Source data are provided as a Source Data file.


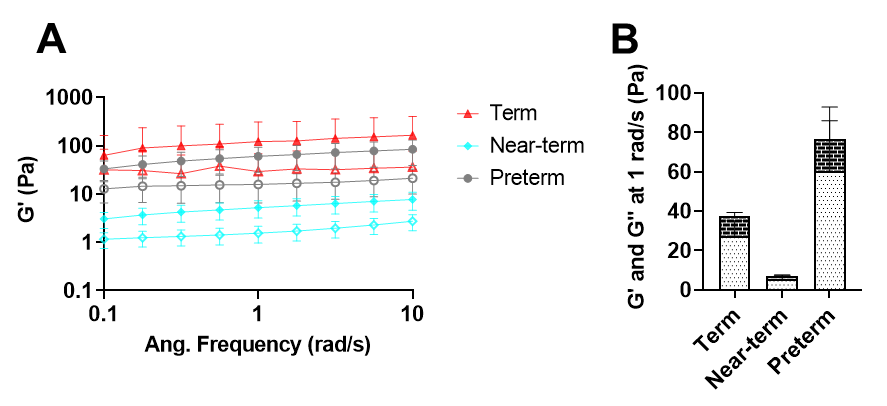


**Fig. S4**. **Effect of birth maturity on elasticity and intermolecular interactions of *ex vivo* small intestinal mucus of from 5 day old healthy piglets.** **A)** Storage modulus (G') and loss modulus (G'') of mucus as a function of increasing angular frequency, using an oscillation stress of 0.1 Pa, well within the linear viscoelastic range. **B)** Difference between G' and G'' obtained from **A** at 1 rad/s. G' is shown as filled symbols in **(A)** and dotted bars in **(B)**, whereas G'' is shown as open symbols in **(A)** and bricked bars in **(B)**. Mucus was collected from healthy small intestine from 5 day old term, near-term or preterm born piglets. All data are presented as means with standard deviations (N=3–4, n=1). For **B)** one outlier was identified for the term group according to the outlier analysis and not included in the depicted data. Source data are provided as a Source Data file.

**Fig. S5**. **Effect of birth maturity on the viscosity of *ex vivo* small intestinal mucus of from 5 day healthy piglets.** Viscosity of mucus with increasing shear rate using a continous flow step. Mucus was collected from healthy small intestine from 5 day old term, near-term or preterm born piglets. All data are presented as means with standard deviations (N=3–4, n=1). Source data are provided as a Source Data file.


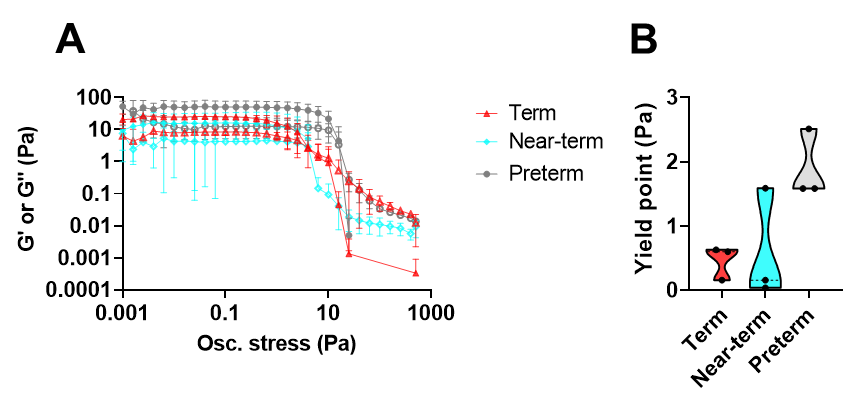


**Fig. S6**. **Effect of birth maturity on stress stability of *ex vivo* small intestinal mucus of from 5 day healthy piglets.** **A)** Storage modulus (G') and loss modulus (G'') of mucus as a function of increasing oscillatory stress with a constant angular frequency of 1 rad/s. **B)** Yield point ( the oscillatory stress needed for 5 % decrease of G') obtained from **A**. G' is shown as filled symbols and G'' as open symbols. Mucus was collected from healthy small intestine from 5 day old term, near-term or preterm born piglets. All data are presented as means with standard deviations (N=3, n=1). The black circles indicate data obtained from individual piglets. Source data are provided as a Source Data file.


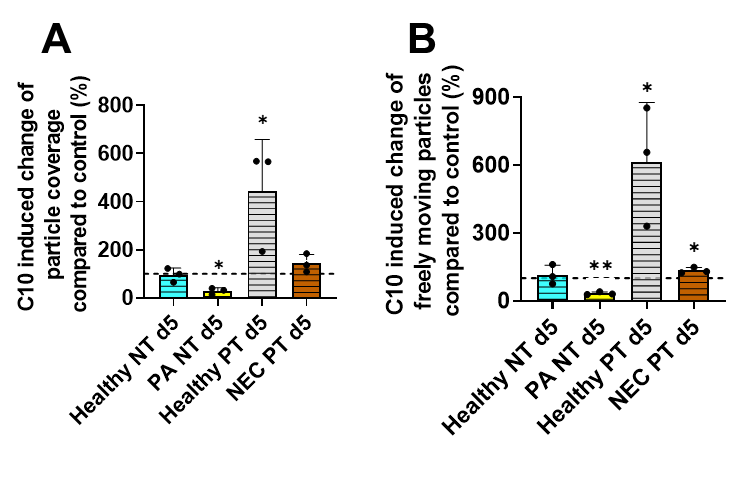


**Fig. S7**. **Effect of 25 mM sodium decanoate (C10) supplementation on the nanoparticle movement in *ex vivo* small intestinal mucus collected postnatal day 5 from either healthy, perinatal asphyxia (PA) or necrotizing enterocolitis (NEC) piglets.** C10 induced change of **A)** average (98% quantile) nanoparticle coverage and **B)** fraction of freely moving 100–300 nm polystyrene nanoparticles in mucus obtained with single particle tracking. The black circles indicate data obtained from individual piglets. The dotted line at 100 % indicates the values obtained in absence of C10. Data are presented as means with standard deviation (N=3, n=2). Significantly different data from that obtained in absence of C10 are indicated as follows:* (p<0.05), ** (p<0.01), *** (p<0.001) or **** (p<0.0001). Exact p-values are listed in Supplementary information Tab. S4. Birth maturity of the 5 day old (d5) piglets were either near-term (NT) or preterm (PT). Source data are provided as a Source Data file


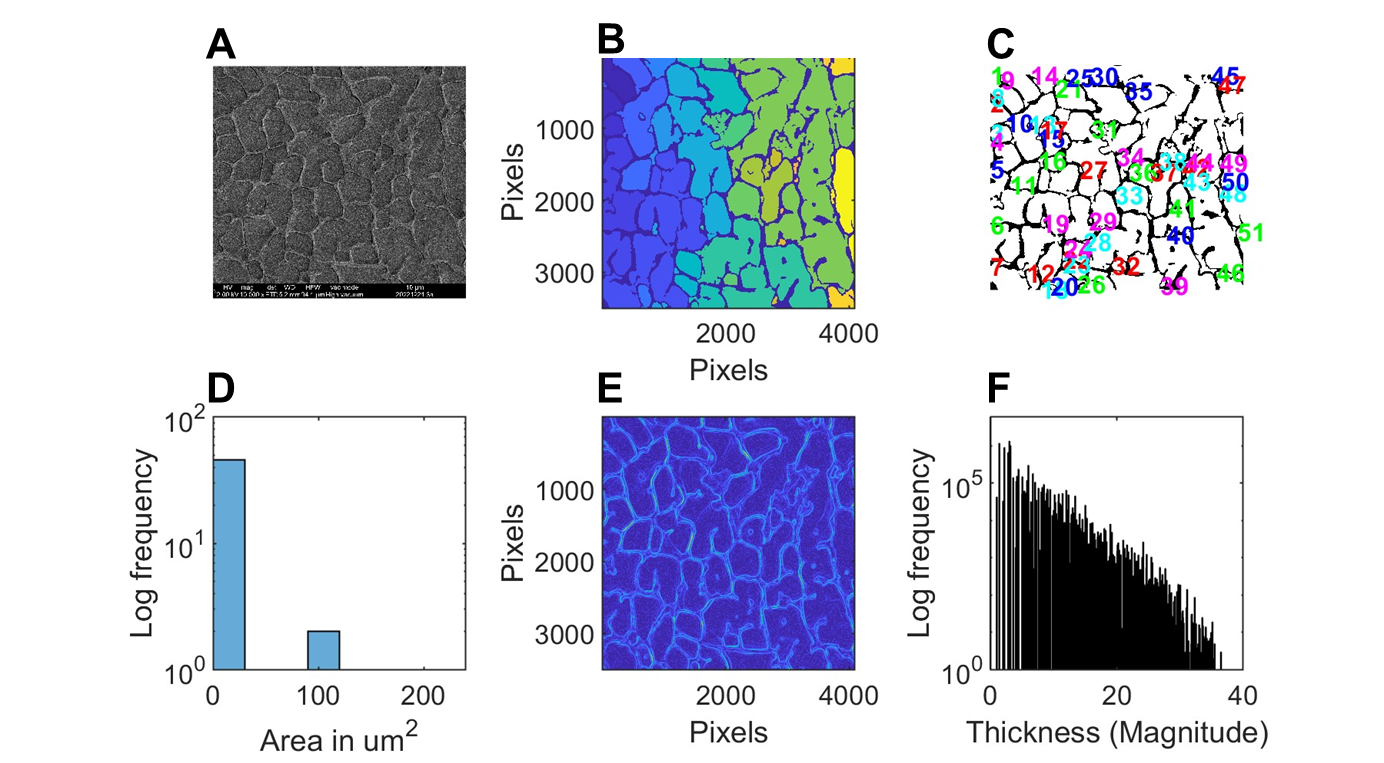


**Fig. S8**. **Data analysis example of cryo scanning electron microscopy (cryo-SEM) image.** **A)** Original cryo-SEM image, **B)** color visualization of identified pores, **C)** number allocation to identified pores marked in white, **D)** distribution of calculated pore areas, **E)** identified pore walls highlighted in blue and **F)** frequency of pore walls upon image sweep of the cryo-SEM images of *ex vivo* porcine intestinal mucus collected from 5 day near-term healthy piglets.

**Tab. S1**. **Effect of postnatal age of term and preterm piglets on diffusion in mucus.** Diffusion coefficients of 100–300 nm polystyrene nanoparticles obtained using single particle tracking in mucus from piglets with different postnatal age and birth maturity (N=3; n=2). All data are presented as means with standard deviation. Source data are provided as a Source Data file.

|  | Term | | | Preterm | |
| --- | --- | --- | --- | --- | --- |
| Postnatal age (days)  [number of particles tracked] | 5  [13649] | 9  [15175] | 19  [4781] | 5  [11495] | 8  [5153] |
| Diffusion coefficients (×10^-2^ µm^2^·s^-1^) | 5.7 ± 1.2 | 1.6 ± 0.4 | 15.0 ± 2.7 | 3.5 ± 1.0 | 12.3 ± 1.5 |

**Tab. S2**. **Fold change of apparent permeability coefficients (P_APP_) of ^3^H-metoprolol, fluorescein-isothiocyanate dextran 4 kDa (FD4) and fluorescein-isothiocyanate dextran 59–77 kDa (FD70) compared to the P_APP_ of ^14^C-mannitol through *ex vivo* small intestinal mucus and mucosa from 5 day term, near-term and preterm piglets.** All data are presented as means with standard deviation (N=1, n=3–6 for mucus and N=3–6, n=1–2 for mucosa). One outlier was identified for FD4 through the mucosa of near-term piglets according to the outlier analysis and not included in the presented data. Statistically significant are indicated as follows: * (p<0.05), ** (p<0.01) or **** (p<0.0001) (red stars: statistically significant from term piglets; blue stars: statistically significant from near-term piglets. Exact p-values are listed in Supplementary information Tab. S4. Source data are provided as a Source Data file.

|  | Mucus | | | Mucosa | | |
| --- | --- | --- | --- | --- | --- | --- |
|  | Term | Near-term | Preterm | Term | Near-term | Preterm |
| Metoprolol | 1.8±0.1 | 1.4±0.1  **** | 1.3±0.1  **** | 2.2±0.8 | 1.6±0.7 | 6.3±8.3 |
| FD4 | 2.0±0.3 | 1.7±0.1 | 1.1±0.1  **/** | 129.4±110.7 | 177.8±77.1 | 576.5±482.7 |
| FD70 | Na | 3.6±0.7* | 6.8±4.1* | Na | Na | 1502.1±244.7 |

**Tab. S3**. **Primer sequences and scources for used gene targets.**

| Protein | Gene | Forward sequence (5’–3’) | Reverse sequence (5’–3’) | Ref |  |
| --- | --- | --- | --- | --- | --- |
| β_2_-Microglobulin | *B2M* | CCCCGAAGGTTCAGGTTTAC | CGGCAGCTATACTGATCCAC | [1] | |
| Succinate dehydrogenase subunit | *SDHA* | CAAACTCGCTCCTGGACCTC | CCGGAGGATCTTCTCACAGC | [1] | |
| Interleukin-6 | *IL6* | CCCACCACAAATGCCGGCCT | GAGGGAATGCCCGTGGACGG | [2] | |
| Interleukin-8 | *IL8* | GGTCTGCCTGGACCCCAAGGAA | TGGGAGCCACGGAGAATGGGTT | [1] | |
| Mucin 1 | *MUC1* | GGTACCCGGCTGGGGCATTG | GGTAGGCATCCCGGGTCGGA | [2] | |
| Mucin 2 | *MUC2* | CTGCTCCGGGTCCTGTGGGA | CCCGCTGGCTGGTGCGATAC | [2] | |
| Claudin-2 | *CLDN2* | TAGGCTACATCCTGGGCCTT | ACGTAAGAACTCGTTCGCCA | [3] | |
| Occludin | *OCLN* | CAGGTGCACCCTCCAGATTG | CAGCGGGTCACCTGATCTTC | [3] | |
| Zonula Occludens | *ZO1* | ACAGTGCCCAGAGACCAAGA | CATTTCCTCGGGGTAGGGGT | [3] | |

**Tab. S4**. **P-values and type of statistical test for comparison of sample values shown in figures.** Stars indicate the stastically significant level used for depiction in the figures. P_APP_: apparent permeability coefficient. d: postnatal day. FD4: fluorescein-isothiocyanate dextran 4 kDa. FD70: fluorescein-isothiocyanate dextran 59–77 kDa. NT: near-term. PT: preterm TMCT: Tukey’s multiple comparisons test. T-test was unpaired unless stated. BFW: Brwon-Forsythe and Welch. Welch’s and BFW correction was used when the group did not have equal standard deviation.

| Figure | Condition | Variables | Test | p-value |
| --- | --- | --- | --- | --- |
| 1B | Goblet cell density | d5 vs d9 vs d19 | Linear model | <0.001*** |
| 1C | Mannitol P_APP_ values | d5 vs d8 | Welch | 0.1382 |
|  | Metoprolol P_APP_ values | d5 vs d8 | T-test | 0.6433 |
|  | FD4 P_APP_ values | d5 vs d8 | Welch | 0.0019** |
|  | FD70 P_APP_ values | d5 vs d8 | T-test | 0.0339* |
| 1D | Fraction of freely moving nanoparticles | Term d5 vs d9 vs d19 | One-way ANOVA  TMCT d5 vs d9  TMCT d5 vs d19  TMCT d9 vs d19 | 0.0015**  0.0092**  0.0013**  0.1570 |
|  | Fraction of freely moving nanoparticles | PT d5 vs d8 | T-test | 0.0007*** |
| 1E | Coverage of nanoparticles | Term d5 vs d9 vs d19 | One-way ANOVA  TMCT d5 vs d9  TMCT d5 vs d19  TMCT d9 vs d19 | 0.0841  0.0727  0.3397  0.4806 |
|  | Coverage of nanoparticles | PT d5 vs d8 | T-test | 0.0110* |
| 3B | Mucus mannitol P_APP_ values | Term vs NT vs PT | One-way ANOVA  TMCT term vs NT  TMCT term vs PT  TMCT NT vs PT | <0.0001****  <0.0001****  <0.0001****  0.0097** |
|  | Mucus metoprolol P_APP_ values | Term vs NT vs PT | One-way ANOVA  TMCT term vs NT  TMCT term vs PT  TMCT NT vs PT | <0.0001****  <0.0001****  <0.0001****  0.0003*** |
|  | Mucus FD4 P_APP_ values | Term vs NT vs PT | One-way ANOVA  TMCT term vs NT  TMCT term vs PT  TMCT NT vs PT | <0.0001****  0.0019**  <0.0001****  <0.0001**** |
|  | Mucus FD70 P_APP_ values | NT vs PT | T-test | 0.2247 |
|  | Mucosa mannitol P_APP_ values | Term vs NT vs PT | One-way ANOVA  TMCT term vs NT  TMCT term vs PT  TMCT NT vs PT | 0.0339*  0.2722  0.0272*  0.3671 |
|  | Mucosa metoprolol P_APP_ values | Term vs NT vs PT | One-way ANOVA  TMCT term vs NT  TMCT term vs PT  TMCT NT vs PT | 0.1945  0.1736  0.3625  0.8499 |
|  | Mucosa FD4 P_APP_ values | Term vs NT vs PT | One-way ANOVA  TMCT term vs NT  TMCT term vs PT  TMCT NT vs PT | 0.9440  0.9515  0.9619  0.9994 |
| 4B | G' at 1 rad/s | Term vs NT vs PT | One-way ANOVA  TMCT term vs NT  TMCT term vs PT  TMCT NT vs PT | 0.0352*  0.4014  0.1720  0.0302* |
| 4C | Loss factor at 1 rad/s | Term vs NT vs PT | One-way ANOVA  TMCT term vs NT  TMCT term vs PT  TMCT NT vs PT | 0.0013**  0.0133*  0.0011**  0.0768 |
| 4D | Viscosity at 0.4 s^-1^ | Term vs NT vs PT | BFW-ANOVA  TMCT term vs NT  TMCT term vs PT  TMCT NT vs PT | 0.0145*  0.2018  0.5752  0.0283* |
| 4E | Flow point | Term vs NT vs PT | One-way ANOVA  TMCT term vs NT  TMCT term vs PT  TMCT NT vs PT | 0.0087**  0.8117  0.0206*  0.0103* |
| 5B | Freely moving particles | NT healthy vs PA | T-test | 0.0541 |
|  | Freely moving particles | PT healthy vs NEC | T-test | 0.0005*** |
|  | Diffusion coefficient | NT healthy vs PA | T-test | 0.1952 |
|  | Diffusion coefficient | PT healthy vs NEC | Welch’s T-test | 0.0288* |
| 5C | Elasticity | NT healthy vs PA | T-test | 0.0103* |
|  | Viscosity | NT healthy vs PA | T-test | 0.4157 |
| 5D | PA mucus mannitol P_APP_ values | NT healthy vs PA | T-test | 0.1242 |
|  | PA mucus metoprolol P_APP_ values | NT healthy vs PA | T-test | 0.0033** |
|  | PA mucus FD4 P_APP_ values | NT healthy vs PA | T-test | 0.1958 |
|  | PA mucus FD70 P_APP_ values | NT healthy vs PA | T-test | 0.2062 |
|  | NEC mucus mannitol P_APP_ values | PT healthy vs NEC | T-test | 0.0144* |
|  | NEC mucus etoprolol P_APP_ values | PT healthy vs NEC | T-test | <0.0001**** |
|  | NEC mucus FD4 P_APP_ values | PT healthy vs NEC | T-test | 0.0258* |
|  | NEC mucus FD70 P_APP_ values | PT healthy vs NEC | T-test | 0.9735 |
|  | PA mucosa mannitol P_APP_ values | NT healthy vs PA | T-test | 0.5078 |
|  | PA mucosa metoprolol P_APP_ values | NT healthy vs PA | T-test | 0.6239 |
|  | PA mucosa FD4 P_APP_ values | NT healthy vs PA | Welch’s T-test | 0.2273 |
|  | NEC mucosa mannitol P_APP_ values | PT healthy vs NEC | T-test | 0.3700 |
|  | NEC mucosa etoprolol P_APP_ values | PT healthy vs NEC | T-test | 0.6083 |
|  | NEC mucosa FD4 P_APP_ values | PT healthy vs NEC | T-test | 0.0223* |
|  | NEC mucosa FD70 P_APP_ values | PT healthy vs NEC | T-test | 0.5684 |
| 6C | Goblet cell density | PT healthy vs NEC | Linear mixed model | 0.057 |
| 6D | FISH bacterial score | PT healthy vs NEC | Nonparametric Spearman correlation | 0.0003*** |
| 6E | *IL6* | PT healthy vs NEC | Linear mixed model | <0.001*** |
|  | *IL8* | PT healthy vs NEC | Linear mixed model | <0.05* |
|  | *MUC1* | PT healthy vs NEC | Mann Whitney test | 0.217 |
|  | *MUC2* | PT healthy vs NEC | Mann Whitney test | 0.585 |
|  | *CLDN2* | PT healthy vs NEC | Linear mixed model | <0.001*** |
|  | *OCLN* | PT healthy vs NEC | Mann Whitney test | 0.255 |
|  | *ZO1* | PT healthy vs NEC | Linear mixed model | 0.218 |
| 7A | Coverage | NT healthy control vs +C10 | Paired T-test | 0.6604 |
|  | Coverage | PT healthy control vs +C10 | Paired T-test | 0.0383* |
|  | Coverage | NT PA control vs +C10 | Paired T-test | 0.0239* |
|  | Coverage | PT NEC control vs +C10 | Paired T-test | 0.1948 |
| 7C | Diffusion coefficient | NT healthy control vs +C10 | Paired T-test | 0.1416 |
|  | Diffusion coefficient | PT healthy control vs +C10 | Paired T-test | 0.0170* |
|  | Diffusion coefficient | NT PA control vs +C10 | Paired T-test | 0.0411* |
|  | Diffusion coefficient | PT NEC control vs +C10 | Paired T-test | 0.2446 |
| 7D | Mannitol P_APP_ values | NT healthy control vs +C10 | T-test | 0.0130* |
|  | Mannitol P_APP_ values | PT healthy control vs +C10 | T-test | 0.0112* |
|  | Mannitol P_APP_ values | NT PA control vs +C10 | T-test | 0.0910 |
|  | Mannitol P_APP_ values | PT NEC control vs +C10 | T-test | 0.0613 |
|  | Metoprolol P_APP_ values | NT healthy control vs +C10 | T-test | <0.0001**** |
|  | Metoprolol P_APP_ values | PT healthy control vs +C10 | T-test | <0.0001**** |
|  | Metoprolol P_APP_ values | NT PA control vs +C10 | T-test | <0.0001**** |
|  | Metoprolol P_APP_ values | PT NEC control vs +C10 | T-test | 0.0001*** |
|  | FD4 P_APP_ values | NT healthy control vs +C10 | T-test | 0.0032** |
|  | FD4 P_APP_ values | PT healthy control vs +C10 | Welch’s T-test | 0.0504 |
|  | FD4 P_APP_ values | NT PA control vs +C10 | T-test | 0.0115* |
|  | FD4 P_APP_ values | PT NEC control vs +C10 | Welch’s T-test | 0.0652 |
|  | FD70 P_APP_ values | NT healthy control vs +C10 | T-test | 0.0998 |
|  | FD70 P_APP_ values | PT healthy control vs +C10 | T-test | 0.1125 |
|  | FD70 P_APP_ values | NT PA control vs +C10 | T-test | 0.2575 |
|  | FD70 P_APP_ values | PT NEC control vs +C10 | T-test | 0.0896 |
| 7E | Pore size | NT healthy control vs +C10 | T-test | 0.0219* |
|  | Pore size | PT healthy control vs +C10 | Welch’s T-test | 0.0026** |
|  | Pore size | NT PA control vs +C10 | T-test | 0.0215* |
|  | Pore size | PT NEC control vs +C10 | Welch’s T-test | 0.0004*** |
| 7F | Wall area | NT healthy control vs +C10 | T-test | 0.0007*** |
|  | Wall area | PT healthy control vs +C10 | T-test | 0.0066** |
|  | Wall area | NT PA control vs +C10 | T-test | 0.2741 |
|  | Wall area | PT NEC control vs +C10 | Welch’s T-test | <0.0001**** |
| S6B | Yield point | Term vs NT vs PT | One-way ANOVA  TMCT term vs NT  TMCT term vs PT  TMCT NT vs PT | 0.0506  0.9610  0.0621  0.0872 |
| S7A | Coverage | NT healthy control vs +C10 | Paired T-test | 0.6604 |
|  | Coverage | PT healthy control vs +C10 | Paired T-test | 0.0383* |
|  | Coverage | NT PA control vs +C10 | Paired T-test | 0.0239* |
|  | Coverage | PT NEC control vs +C10 | Paired T-test | 0.1948 |
| S7B | Freely moving particles | NT healthy control vs +C10 | Paired T-test | 0.6408 |
|  | Freely moving particles | PT healthy control vs +C10 | Paired T-test | 0.0142* |
|  | Freely moving particles | NT PA control vs +C10 | Paired T-test | 0.0018** |
|  | Freely moving particles | PT NEC control vs +C10 | Paired T-test | 0.0268* |

**References**

1. Tudela, C. V., Boudry, C., Stumpff, F., Aschenbach, J. R., Vahjen, W., et al. Down-regulation of monocarboxylate transporter 1 (MCT1) gene expression in the colon of piglets is linked to bacterial protein fermentation and pro-inflammatory cytokine-mediated signalling. *Br. J. Nutr.* **113**, 610-7 (2015).

2. Pieper, R., Kröger, S., Richter, J. F., Wang, J., Martin, L., et al. Fermentable fiber ameliorates fermentable protein-induced changes in microbial ecology, but not the mucosal response, in the colon of piglets. J. Nutr. **142**, 661-7 (2012).

3. Pieper, R., Scharek-Tedin, L., Zetzsche, A., Röhe, I., Kröger, S., et al. Bovine milk–based formula leads to early maturation-like morphological, immunological, and functional changes in the jejunum of neonatal piglets. J. Anim. Sci. **94**, 989-99 (2016).
